# Water Quality of U.S. Drinking Water Kiosks: Lead Release from “Lead-free” Plumbing after Reverse Osmosis Treatment

**DOI:** 10.1021/acs.est.5c10647

**Published:** 2026-02-11

**Authors:** Samantha Zuhlke, Drew E. Latta, Kate Beeman, Amukta Gantalamohini, James Kacer, Grace Koch, Danielle Land, Abby McKeone, Casie A. Meyer, Matthew R. Nagorzanski, Abdul H. Quraishi, LilliAnna Scott, Hanseob Shin, Martin A. St Clair, Darrin A. Thompson, David M. Cwiertny

**Affiliations:** † School of Planning and Public Affairs, 4083 University of Iowa, Iowa City, Iowa 52242, United States; ‡ IIHR-Hydroscience and Engineering, Department of Civil & Environmental Engineering, University of Iowa, Iowa City, Iowa 52242, United States; § Center for Health Effects of Environmental Contamination (CHEEC), University of Iowa, Iowa City, Iowa 52242, United States; ∥ Department of Civil & Environmental Engineering, University of Iowa, Iowa City, Iowa 52242, United States; ⊥ C.S. Mott Department of Public Health, Michigan State University, Flint, Michigan 48502, United States; # State Hygienic Laboratory of Iowa, University of Iowa, Coralville, Iowa 52241, United States; ∇ Department of Chemistry, University of Iowa, Iowa City, Iowa 52242, United States

**Keywords:** water quality, drinking water, commercial water, water vending machines, public policy

## Abstract

Many Americans distrust tap water, leading them to purchase more expensive drinking water sold from water vending machines (e.g., kiosks) that are poorly regulated and sparingly monitored for quality. Here, we analyzed the water quality of 20 kiosks from 4 different manufacturers across 6 states in the first comprehensive comparison of the chemical and microbial characteristics of kiosk water to paired tap samples. Of the 16 kiosks listing specific water treatment processes (others indicated “filtered” or “purified”), only 1 dispensed water with ionic composition (e.g., Na^+^, Ca^2+^, Mg^2+^) inconsistent with the stated treatment. Most kiosks tested used reverse osmosis (RO), which removed fluoride and residual disinfectant, although we found no evidence of microbial contamination. RO also provided the benefit of removing per- and polyfluoroalkyl substances. However, we frequently detected higher lead levels in kiosk water than in nearby tap water. Lead was detected (>0.05 μg/L, our method detection limit) in 15 kiosks; 5 were >1 μg/L (American Academy of Pediatrics recommendation), 2 were >5 μg/L (FDA allowable level for bottled water), and 1 was >10 μg/L (US EPA Action Level). Lead co-occurred with zinc and copper, consistent with corrosion of lead-containing plumbing materials. XRF analysis of plumbing in 2 kiosks from different manufacturers with nationwide distribution confirmed this suspicion although all components in question met the definition of lead free under the Safe Drinking Water Act. Lead release was most evident with the use of RO treatment, which can produce corrosive water low in alkalinity and pH. Going forward, the removal of lead-containing plumbing components downstream of RO treatment and regulation with routine testing of kiosk water quality is imperative to address this unchecked public health risk.

## Graphical abstract

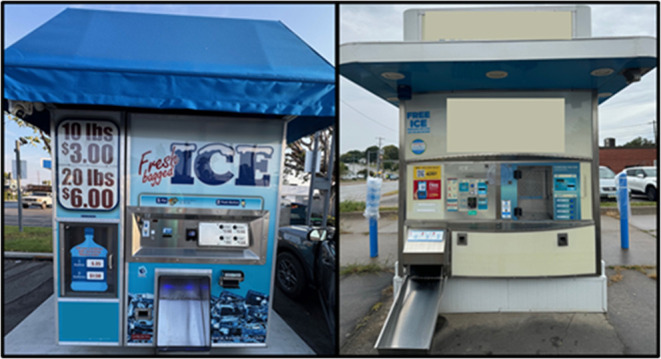

## Introduction

Water kiosks are privately owned, drinking water vending machines that dispense water in gallon units, typically priced at $0.25–0.35 per gallon (i.e., roughly 7000% more than a gallon of tap water in most major U.S. cities).^1^ Kiosks often market themselves as offering a product that is safer than tap water,^1,2^ and many historically marginalized Americans purchase commercial drinking water, in part due to the distrust of tap water.^1–7^ Nationwide, kiosks are more likely to be located in nonwhite, lower socioeconomic areas.^1^ Many kiosks source their water from the local municipal water supply and then claim to employ additional advanced treatment processes like reverse osmosis (RO) to remove impurities or contaminants from the tap water (e.g., ref 8). Despite these claims, the quality of drinking water sold by kiosks is unknown due to poor regulation and sparse monitoring of these systems. Limited research finds that kiosk water can contain indicators of microbial contamination,^9–12^ but to date, no studies have examined how the overall water quality of kiosk water relates to U.S. drinking water standards.

Tap water in the U.S. is regulated by the Safe Drinking Water Act (SDWA) under the EPA, but the SDWA’s application to water kiosks is unclear. The EPA defines kiosks as noncommunity water systems,^13^ which are classified as either “nontransient” or “transient” systems. Confoundingly, some sources identify kiosks as transient systems^14^ and others as nontransient systems.^15^ This lack of clarity produces conflicting regulatory regimes, overseeing kiosks from state to state with insufficient enforcement. At the federal level, kiosk water quality is rarely reported on in the Safe Drinking Water Information System (SDWIS).^16^ At the state level, one California-based investigation demonstrated that kiosks are underinspected and frequently violate state code.^17,18^ This lack of oversight means kiosk operators are key to maintaining kiosk water quality.^14^ However, another California-based study found over 27% of kiosks surveyed did not have a last date of service posted and 17% did not post operator contact information.^12^

Here, we undertook the first comprehensive investigation of kiosk drinking water quality, including the first examination of metals and perfluoroalkyl and polyfluoroalkyl substances (PFAS). We collected water samples from 20 kiosks owned and operated by 4 different manufacturers (Figure 1) found in Iowa and 5 surrounding states with a range of stated treatment processes, including reverse osmosis (RO). As a general approach, we analyzed kiosk water samples for their physiochemical parameters, microbiology, and levels of metals and PFAS, which we then compared to a paired tap water sample collected nearby.

**Figure 1:**
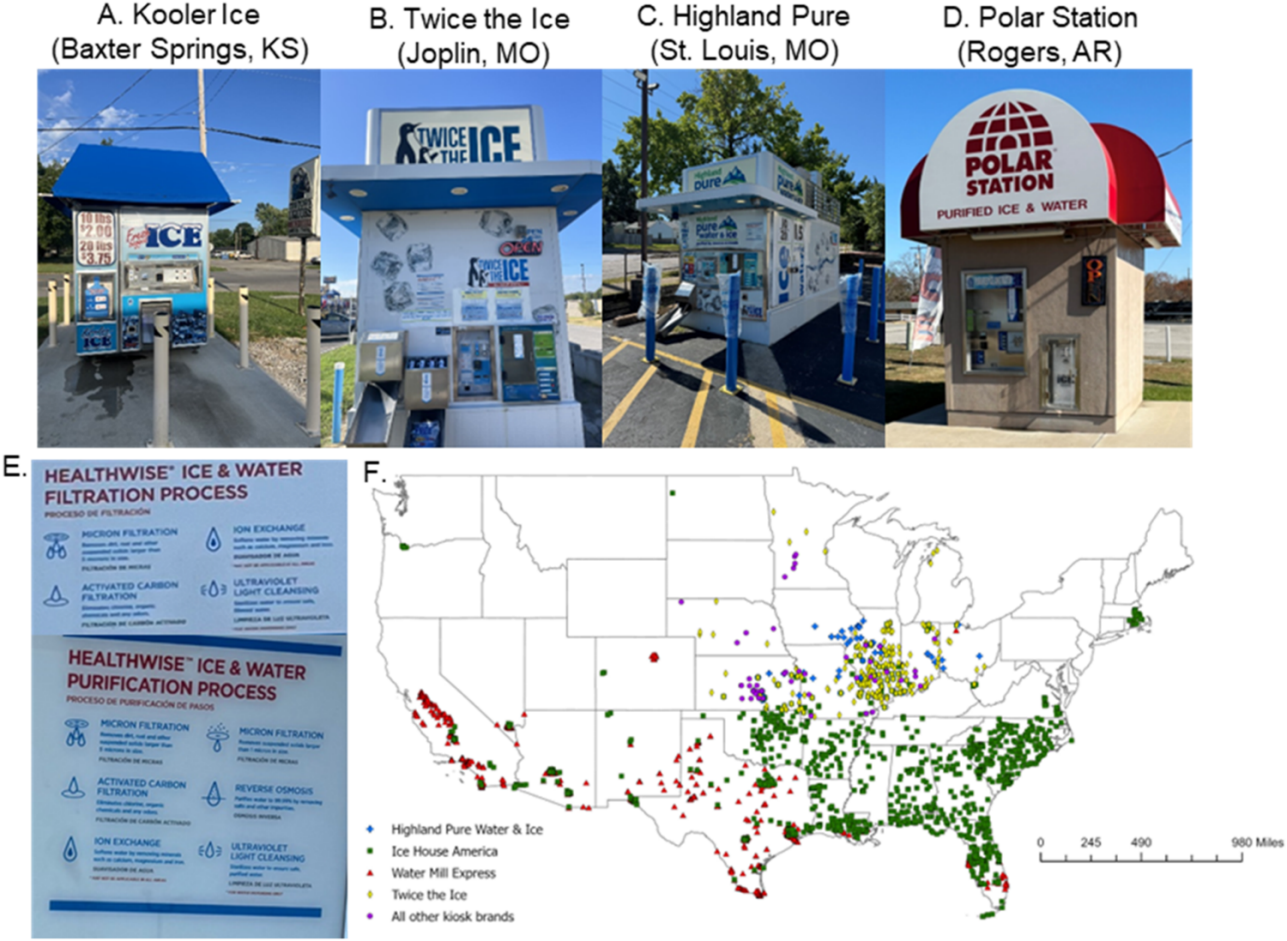
Representative pictures of 4 kiosk manufacturers: (A) Kooler Ice, (B) Twice the Ice, (C) Highland Pure, and (D) Polar Station from which water samples were primarily collected in this study. Also provided are (E) representative images of the signage used to indicate the level of treatment used by kiosks in (top) Joplin, MO, and (bottom) St. Louis, MO. (F) Map of the U.S. drinking water kiosk locations symbolized by company ownership, based on 2017 and 2024 data collection. The number of kiosks is underrepresented, given the exclusion of Kooler Ice kiosks and limited data collection in 2024. *Sources: Photos taken by research team; Original data collected via Google Maps and Google Street View in 2017 and 2024*.

This work significantly expands on limited existing kiosk water quality studies that primarily focus on a single community^10–12^ or a single water quality dimension^19^ like microbial contamination.^10,11^ With reports of PFAS occurrence in other commercial water supplies like bottled water,^20^ it is the first to address whether kiosks represent a viable alternative for drinking water in communities with PFAS contamination. Finally, with the first metals analysis, it fills existing gaps left by prior studies that have reported relatively low pH values in kiosk water that could promote metal corrosion (e.g., Hile et al. reported that 52% of their kiosk water samples had a pH value below 6.5 ^10^). This is particularly important for kiosks using RO treatment because of recent reports illustrating that RO-treated water can be corrosive to plumbing materials without proper stabilization.^21,22^

## Methods

### Sampling Details

Two primary sampling campaigns were conducted in Iowa in December 2023 and in Iowa and surrounding states in September 2024 (a full sampling matrix including kiosk locations, manufacturers, dates tested, and other relevant information is provided in Table S1). For convenience, we hereafter refer to these sampling campaigns as Winter 2023 and Summer 2024, respectively. Kiosk water samples were collected for analysis of microbiological species (only in Winter 2023), PFAS, major ions (e.g., chloride, nitrate), metals (e.g., lead, copper), and bulk water quality characteristics (e.g., pH, alkalinity). In most instances, a field probe was also used to measure pH, temperature, conductivity, and residual chlorine at the time of sample collection. Also in the Summer of 2024, ice (dispensed in a 5 or 10 lbs. plastic bag) was purchased, when available, for analysis. Additional details of the sequence and volumes of kiosk water collected for analysis can be found in Supporting Information (SI).

For comparison, paired tap water samples were collected at a publicly accessible tap adjacent to each kiosk, typically within 1 mile of the kiosk (see Table S1). Details of the community water systems used as source water for Iowa kiosks are listed in Table S2. The sequence and volumes of water collected at all public taps were identical to those used for kiosks, as described in SI. For quality assurance/quality control purposes, field blanks were also collected periodically, as described in SI.

In response to results from these primary sampling campaigns, additional sampling events were conducted between September and December of 2024 (hereafter Fall 2024) and in Spring 2025. These additional sampling activities primarily focused on kiosks outside the state of Iowa in Kansas, Missouri, Arkansas, and Oklahoma, including repeated sampling of select locations from Summer 2024. The locations and vendors of these kiosks are also summarized in Table S1. This included temporal sample collection at one kiosk location (Muscatine, Iowa), in which periodic sampling was collected over a ∼24 h period from 10:30 pm on September 17, 2024, to 6:00 pm on September 18, 2024. Across all sampling events, all kiosks were sampled at least twice, except for those located in Burlington, IA; Moline, IL; Joplin, MO; St. Louis, MO; Rogers, AR; Miami, OK.

### Analytical Methods

All samples were analyzed for the presence of microbes, PFAS, major ions, and metals using the established methods. Details of all sample processing and analysis, including field probes, can be found in SI. Ion chromatography was used to measure chloride, bromide, sulfate, fluoride, and nitrate. We analyzed for 25 PFAS species (Table S3) using a modified version of EPA Method 533.^23^ For metals, we primarily report total metal concentrations that were measured after digestion in 2% (v/v) nitric acid and 0.2% (w/v) hydroxylamine hydrochloride at 50 °C.^24^ In select cases, we also report dissolved metal concentrations, which were measured after filtering the water sample with a 0.45 μm syringe-tip polypropylene filter. Quantification by ICP-MS (Agilent 7900, Agilent Technologies, Inc.) followed U.S. EPA Method 200.8 ^25^ for Cu, Zn, Sn, and Pb with the addition of major cations (Na, Mg, K, and Ca) and total P using similar QA/QC procedures. Microbial analysis included enumeration of heterotrophic bacteria, coliform, *E. coli*, and *Enterococci*, along with DNA extraction for microbiome analysis.

## Results and Discussion

### Comparing General Quality Characteristics of Kiosk and Tap Water

Clear signage indicating specific water treatment processes used by the kiosk was noted in only 16 of the 20 kiosks tested (see Table S1). Of these, 14 kiosks operated by Highland Pure Water & Ice (HP) indicated the use of RO treatment. The other 2 kiosks with clear signage were Twice the Ice (TI) kiosks located in Joplin, MO, and Miami, OK, which indicated micron filtration, activated carbon filtration, ion exchange, and ultraviolet light cleansing. In all locations indicating RO treatment but one, a HP kiosk in Washington, IA, the ionic composition of the purchased water was consistent with expectations for RO treatment (i.e., low levels of total conductivity and dissolved ions; Table 1). For the HP kiosk in Washington, IA, the ionic content of the purchased water was nearly identical to the corresponding tap water except for lower levels of hardness causing ions, Ca^2+^ and Mg^2+^, consistent with only the use of ion (cation) exchange for water softening. For water dispensed from the TI kiosk in Joplin, MO (Table 1), ionic composition was also consistent with softening (e.g., low Ca^2+^ and Mg^2+^), in agreement with expectations from the kiosk’s signage. A corresponding tap water sample was not collected in Miami, OK, preventing a performance assessment of that TI kiosk, although Ca^2+^ and Mg^2+^ levels in the kiosk water were higher than those observed in TI kiosk water from Joplin, MO.

**Table 1:** Locations and Vendors of Sampled Kiosks, Including Relevant Water Quality Data from Summer and Fall 2024 (Unless Date Otherwise Noted)^a^

						anions (mg/L)				cations (mg/L)^b^		
location and vendor^c^	sample	*T* (°C)	field pH	alkalinity (mg/L CaCO_3_)	conductivity (lab) (μS/cm)	F^–^	Cl^–^	NO_3_^–^	SO_4_^2–^	Na^+^	Mg^2+^	Ca^2+^
Clinton, IA	tap	19.5	7.4	268	758	0.46	42.4	0.02	43.4	48.46	21.06	54.48
HP	kiosk	14.4	6.53	<MDL^d^	63	0.03	3.9	DET^e^	2.0	6.63	1.34	3.05
Mt. Pleasant, IA	tap	22.8	7.77	154	789	1.18	74.8	0.06	95.0	119.50	7.71	16.61
HP	kiosk	18.3	6.30	<MDL	33	0.03	4.2	0.02	0.4	5.66	0.02	<0.053
Ft. Madison, IA	tap	22	7.86	113	355	0.52	8.9	DET	40.3	13.30	9.45	34.91
HP	kiosk	17.8	6.0	<MDL	14	0.02	0.5	DET	0.04	1.95	0.08	0.21
Burlington, IA	tap	21.8	8.97	48	301	0.53	29.8	0.85	41.2	12.78	7.90	20.41
HP	kiosk	16.4	9.38	<MDL	22	0.01	0.9	0.07	0.5	2.53	0.07	0.10
Muscatine, IA	tap	21.9	7.31	201	577	0.47	27.8	1.90	43.5	14.22	21.55	63.64
HP (9/8/24)	kiosk	16.1	6.21	<MDL	123	0.02	2.2	0.65	0.05	3.68	0.27	1.45
HP (11/2/24)	kiosk	10.6	5.86	<MDL	44.8	0.01	2.6	0.5	<MDL	4.17	0.43	2.29
HP (12/6/24)	kiosk	15.1	6.11	<MDL	41.4	0.01	2.6	0.43	<MDL	6.09	0.30	0.84
KI (11/2/24)	kiosk	18.6	7.06	222	649	0.48	26.7	1.3	48.8	12.96	22.50	66.09
KI (3/11/25)	kiosk	NA^f^	6.86^g^	<MDL	67.5	0.03	3.3	0.38	0.74	4.53	2.10	5.10
Bettendorf, IA	tap	20.9	7.36	147	399	0.48	24.6	0.99	49.2	13.93	18.50	40.69
HP	kiosk	21.7	6.10	<MDL	23	0.02	1.8	0.22	0.05	3.24	0.19	0.48
Davenport, IA	tap	21.9	6.75	140	474	0.59	26.2	2.21	51.5	13.59	18.57	42.03
HP1	kiosk	19.1	5.82	<MDL	17	0.02	1.2	0.19	0.03	2.06	0.19	0.67
Davenport, IA	tap	21.2	7.23	134	496	0.53	26.0	1.10	52.5	14.03	18.77	41.83
HP2	kiosk	13.6	5.89	<MDL	18	0.02	1.3	0.25	0.07	2.48	0.13	0.33
Ottumwa, IA	tap	24	9.64	<MDL	321	0.21	40.6	0.85	62.4	19.32	4.50	33.55
HP	kiosk	26.6	9.12	<MDL	28	<MDL	0.4	0.03	0.06	2.34	<0.004	<0.053
Moline, IL	tap	22.1	9.05	58	289	0.65	25.7	0.99	40.4	14.95	5.09	28.60
HP	kiosk	14.6	9.71	<MDL	21	DET	0.9	0.10	0.3	3.69	<0.004	<0.053
Fairfield, IA	tap	22.9	8.65	65	1019	0.70	76.6	0.22	290.2	158.47	2.45	29.78
HP	kiosk	33.6	8.66	<MDL	70	0.07	10.3	0.07	5.3	12.97	<0.004	<0.053
Washington, IA	tap	23.0	8.63	69	472	0.26	23.0	0.03	118.3	51.96	9.17	19.61
HP	kiosk	23.6	8.82	68	477	0.26	23.4	0.03	117.3	67.88	5.80	9.54
Des Moines, IA	tap	22.7	9.20	39	344	0.741	50.1	1.80	49.9	21.57	10.96	27.88
HP	kiosk	17.6	9.55	<MDL	111	<MDL	2.1	0.07	0.4	13.71	0.80	1.20
Baxter Springs, KS	tap	26.5	7.57	133	392	0.9	31.7	0.9	24.8	16.22	4.65	53.63
KI	kiosk	26.4	6.45	<MDL	31	DET	2.0	0.2	0.3	2.42	0.13	1.64
Joplin, MO	tap	36.8	7.59	124	361	0.61	21.9	1.9	7.9	14.96	3.87	50.06
TI	kiosk	29.2	7.64	137	374	0.59	22.6	2.2	8.0	80.44	<0.004	<0.053
St. Louis, MO	tap	NA^f^	9.47^g^	<MDL	803	0.56	60.8	0.5	186.5	52.33	17.67	20.29
HP	kiosk	NA	7.43^g^	42	48	0.03	2.9	0.2	0.9	7.93	0.03	0.06
Neosho, MO	tap	NA	NA	NA	NA	NA	NA	NA	NA	NA	NA	NA
TI	kiosk	29.7	7.33	131	349	0.02	20.2	2.4	8.1	76.00	0.01	0.22
Rogers, AR	tap	NA	8.13^g^	33.4	197.4	0.63	8.2	1.10	27.9	NA	NA	NA
PS	kiosk	NA	8.05^g^	38.3	212	0.63	9.1	0.86	30.1	8.72	1.74	24.31
Miami, OK	tap	NA	NA	NA	NA	NA	NA	NA	NA	NA	NA	NA
TI	kiosk	NA	7.88^g^	94.4	366.8	0.30	38.5	DET	12.4	20.64	14.36	25.15

a Values are representative of the results obtained during repeated sampling events of select kiosks.

b Cation concentrations were measured along with metals in the first liter sample.

c HP = Highland Pure Water & Ice; KI = Kooler Ice; TI = Twice the Ice; PS = Polar Station.

d <MDL = value was below the method detection limit for analysis.

e DET = value was above the MDL but less than the method quantification limit for analysis.

f NA= Values are not available either because the parameter was not measured or an appropriate sample was not collected.

g pH of the sample was measured upon the return of the sample to the laboratory rather than in the field.

The remaining 4 kiosks lacked any clear indication of the specific purification techniques employed, often marketing the water and ice sold as “filtered” or “purified.” They were a TI kiosk in Neosho, MO, 2 Kooler Ice (KI) kiosks in Muscatine, IA, and Baxter Springs, KS, and a Polar Station (PS) kiosk in Rogers, AR. For these kiosks, the analysis of the dispensed water provides some insights into the likely treatment processes being used, if any. For example, the purchased water from the TI kiosk in Neosho, MO, exhibited conductivity that was much higher than that anticipated for RO-treated water. It also contained very low Ca^2+^ and Mg^2+^ with elevated levels (76.0 mg/L) of Na^+^, once again consistent with water softening via ion (cation) exchange. Notably, the Na^+^ levels measured in kiosk water (Table 1) from Neosho and Joplin, MO, which indicated the use of ion exchange, are above the US EPA recommended limit for sodium in drinking water (20 mg/L) for individuals on low sodium diets (<500 mg/day) and above US EPA recommendation (30–60 mg/L) to avoid taste issues.^26^ As another example, while the analysis of the KI kiosk in Baxter Springs, KS, was consistent with the use of RO, the ionic composition of the water from the KI kiosk in Muscatine, IA, in Fall 2024 was nearly identical to that of Muscatine tap water (Table 1). In instances like these, customers pay a premium at kiosks for drinking water that may not be significantly different from tap water, may be less aesthetically pleasing, or have quality concerns for certain consumers based on available public health recommendations.

Other notable observations for RO-treated water included the expected removal of fluoride (Table 1), and we also often observed a considerably lower pH of kiosk water relative to the corresponding tap water. It is recognized that RO membranes can remove charged species including carbonate and bicarbonate, as illustrated by the large decreases in alkalinity we observed for kiosk water, but that neutrally charged carbonic acid (H_2_CO_3_) and aqueous CO_2_ (the sum of which is often referred to as H_2_CO_3_*) can cross RO membranes and acidify the permeate.^27,28^

### Microbial Water Quality

For some RO-treated kiosk water in Iowa, measurements often revealed little to no chlorine residual (Table S4). In Winter 2023, residual levels of total chlorine were below the limit of detection in water dispensed from kiosks in Clinton, Muscatine, and Fort Madison and less than the corresponding value in tap water from kiosks in Des Moines, one location in Davenport, Ottumwa, Mt. Pleasant, and Fairfield. Lower residual chlorine in kiosk water is likely due to the expected breakdown and removal of residual disinfectant present in the tap water source during RO treatment.

Although the loss of disinfectant residual during RO treatment raises concerns about microbial growth within the kiosk plumbing, we did not find evidence of significant microbial contamination in HP kiosks in Iowa. Enumeration of heterotrophic plate counts (HPCs) demonstrated that 3 of the 10 tap water samples from Iowa had 20 MPNs/100 mL, whereas no HPC was measurable in any of the kiosk samples. Further, total coliforms, *E. coli* and *Enterococci*, were not detected in any tap or kiosk water sample (Table S5). DNA extraction and sequencing generally revealed lower microbial abundance in kiosk water relative to tap water; although we were able to extract sufficient DNA for microbiome analysis from all tap water samples, only 6 kiosk samples produced sufficient DNA for microbiome analysis. Although the detection of DNA provides no indication of whether pathogenic cells are alive or viable, microbiome analysis, limited to the genus level, revealed evidence for the possible presence of opportunistic pathogens in both tap and kiosk water samples. Also, it revealed interesting clustering in microbial composition based on source water type (e.g., surface water and groundwater) and shifts in microbial composition through the RO treatment used by kiosks. A more detailed discussion of these microbiome results (Table S6 and Figures S1 and S2) can be found in SI.

### PFAS Removal during Kiosk Treatment

During Winter 2023 and Summer 2024, several of the communities with kiosks also had PFAS in their tap water. From Winter 2023 sampling in Iowa (Figure S3a and Table S7), we found detectable levels [above 1 ppt or 1 ng/L] of total PFAS in tap water samples from Des Moines (5.1 ppt total PFAS), Davenport (2 tap locations at 26.3 and 28.6 ppt), Bettendorf (27.4 ppt), Muscatine (77.9 ppt), and Ottumwa (12.1 ppt), whereas tap water from Clinton, Mt. Pleasant, Fort Madison, and Fairfield had no detectable PFAS. Notably, all water systems with detectable PFAS rely on surface water sources, including the Mississippi River (e.g., Davenport and Bettendorf; see Figure S4), except for Muscatine, which sources water from an alluvial aquifer partially recharged by the Mississippi River. In Summer 2024, we observed very little variation in total PFAS levels in communities previously tested in Winter 2023 **(** Figure S3c and Table S8). In addition, although PFAS was not detected in the tap water of Washington, IA, 44.8 ppt of total PFAS was measured in the tap water of Burlington, IA, which also primarily relies on the Mississippi River (along with three alluvial wells). Outside of Iowa, low-level PFAS was detected in tap water from Joplin, MO (4.7 ppt), Baxter Springs, KS (16.1 ppt), and Moline, IL (22.8 ppt).

We found that kiosks using RO treatment were generally effective in removing most, if not all, PFAS species from the sourced tap water (Figure S3b,d and Tables S7 and S8). For RO-treated kiosk water, we observed only a small amount of PFBA (1.5 ppt) in one sample from the Muscatine HP kiosk in Winter 2023. This corresponds to 98% removal of PFBA during RO treatment (relative to the tap water sample) and removal of all other PFAS species detected in Muscatine tap water to levels below detection. All other RO-treated kiosk samples in Iowa and other states were free of PFAS in Winter 2023 and Summer 2024, including the Muscatine HP kiosk sample from Summer 2024.

In contrast, kiosks that did not employ RO treatment exhibited variable performance and were generally less effective at removing PFAS chemicals. In Joplin, MO, we detected both PFBS (1.1 ppt) and PFPeA (1.7 ppt) in water from the TI kiosk with signage indicating the use of microfiltration, activated carbon filtration, cation exchange, and UV disinfection. Although a small amount of PFOS (1.8 ng/L) was detected in the Joplin tap water sample, PFOS was not detected in the kiosk sample. Likely, the activated carbon filter indicated on the Joplin signage, which typically performs better for the removal of longer chain PFAS species,^29^ is responsible for the removal of PFOS and the breakthrough of shorter chain PFBS and PFPeA. Several PFAS species were also detected in water from the KI kiosk in Muscatine, IA, in Fall 2024, but the total was notably lower than previously observed in Muscatine tap water. No treatment signage was available, and we did not observe a change in dissolved ions (Table 1) consistent with effective RO treatment. Accordingly, it appears that this kiosk may also be using activated carbon treatment, which would not impact dissolved ions but could help reduce the total amount of PFAS present in the kiosk water.

### Presence of Lead in Water Samples Dispensed from Kiosks

Sampling between Winter 2023 and Fall 2024 revealed multiple instances of lead in kiosk water at levels above our method detection limit (MDL of 0.05 μg/L or ppb) and often at levels above those detected in corresponding tap water samples and of concern for public health. During Winter 2023 in Iowa, lead levels in the 5th liter of water dispensed from HP kiosks in Muscatine, one Davenport location, and Bettendorf, IA, exceeded the American Academy of Pediatrics (AAP) recommendation of 1 ppb (Figure 2a and Table S9). At Muscatine, the concentration of 9.23 ppb exceeded the U.S. Food and Drug Administration (FDA) limit for bottled water of 5 ppb, was higher than the 90th percentile sample reported by the local municipality for compliance testing,^30^ and approached the recently adopted US EPA Action Level of 10 ppb in the Lead and Copper Rule Improvements (LCRI).^31^

**Figure 2:**
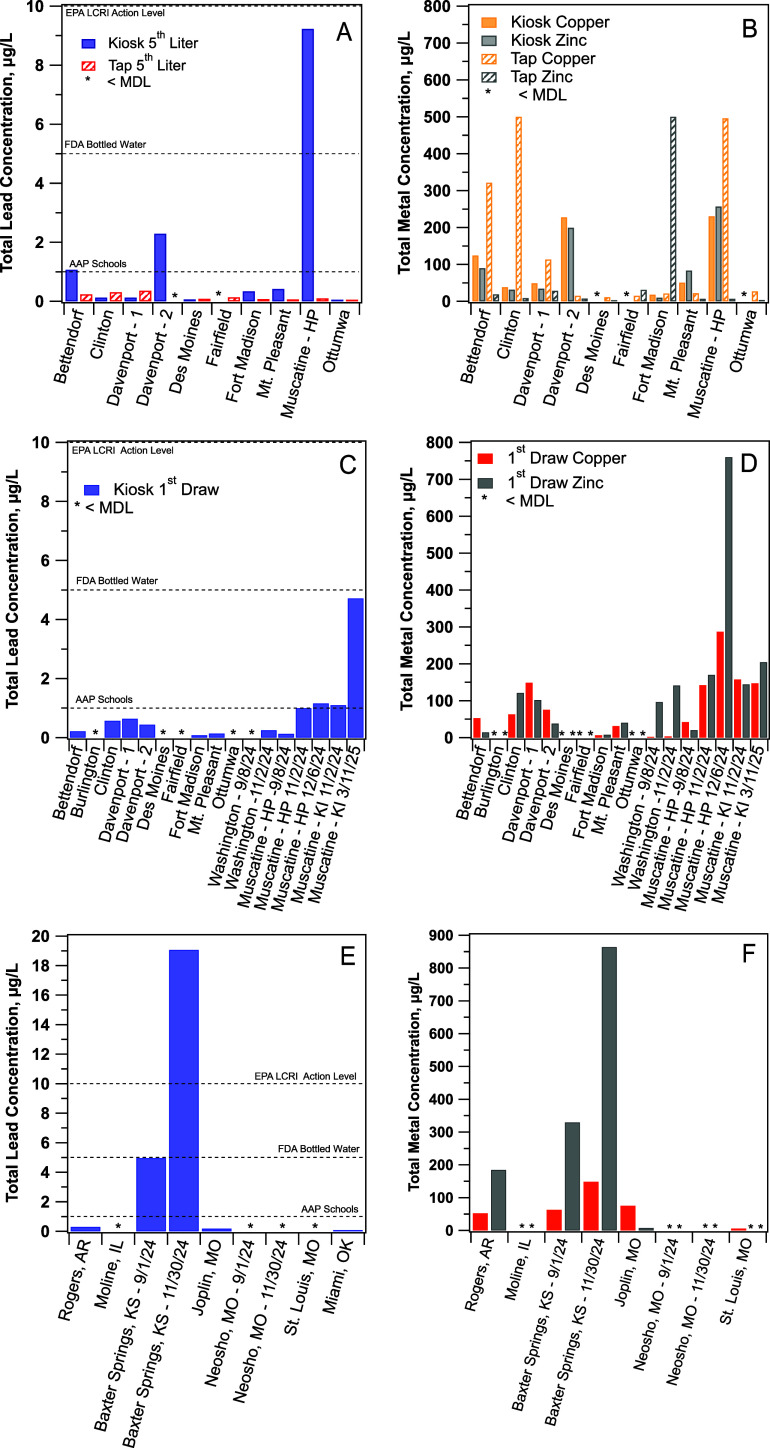
(A) Lead and (B) copper and zinc in the 5th liter of paired kiosk and tap water samples collected during Winter 2023 sampling in Iowa. (C) Lead and (D) copper and zinc in the 1st liter of kiosk water samples collected between Summer 2024 and Spring 2025 in Iowa. (E) Lead and (F) copper and zinc and in the 1st liter of kiosk samples collected in surrounding states during Summer and Fall 2024. The method detection limit for lead is 0.05 μg/L.

These instances of lead detection in kiosk water are concerning because we routinely observed consumers purchasing 5-gallon jugs with the intent of using this water for drinking and food preparation.^32^ For example, these lead concentrations would be of serious concern if kiosk water, purchased under the assumption from marketing claims that it is safer than tap water, is used for the preparation of infant formula. According to the US EPA,^33^ formula-fed infants can receive 40–60% of their total lead exposure from drinking water. Although direct evidence linking specific water lead levels to IQ loss in formula-fed infants is limited in the peer-reviewed literature, studies in children aged 1–5 years demonstrate that each 1 ppb increase in water lead is associated with a 35% increase in BLLs over extended exposure periods.^34^ Importantly, BLLs even below 10 μg/dL are associated with decreased IQ, with the steepest dose–response occurring at the lowest exposure levels.^35,36^

The presence of lead in HP kiosk water was mirrored by copper and zinc (Figure 2b), metals that are also likely plumbing derived. Although the observed copper and zinc levels present no human health hazard, they are unexpected given the use of RO treatment, which should effectively remove lead, copper, and zinc.^37^ Complete lead, copper, and zinc data are tabulated in SI (Table S9).

Lead levels in kiosk water appear to vary with season, likely because of kiosks’ greater use in hotter summer months. When resampling Iowa locations in Summer 2024, we frequently encountered customers during sample collection. We collected both the first and fifth liter of dispensed kiosk water in accordance with LCRI sampling protocols (see SI); at the time, we lacked insights into the configuration of treatment and plumbing inside the kiosk, and the collection of the first and fifth liter provided the easiest way to directly compare the quality of kiosk water to the quality of corresponding tap samples. We presumed that the first liter reflects water in contact with fixtures and fittings near the kiosk outlet (as in LCRI residential sampling), whereas the fifth liter represents flushed water likely originating from the kiosk reservoir. We observed much lower lead concentrations during this round of sampling, with all but one sample having lead levels in the first liter sample less than 1 ppb (Figure 2c). Lead levels also decreased in the fifth liter sample (Table S10). Nevertheless, we continued to see elevated concentrations of zinc and copper (Figures 2d and Table S10) in most kiosk samples, which is indicative of metal corrosion.

Stagnation of water in contact with lead-containing plumbing is known to increase the resulting lead concentration in water.^38^ We suspect, therefore, that the greater use of kiosks in the summer months may limit stagnation, thereby keeping the lead levels in the dispensed water lower. Results from subsequent temporal sampling (Figure S5) of the Muscatine HP kiosk are consistent with a connection between stagnation time and lead levels; lead levels were highest early in the morning after overnight stagnation and subsequently decreased from the first to the fifth liter of water dispensed from the kiosk.

The presence of lead in kiosk water appears to be a problem nationwide for certain manufacturers. In testing conducted outside of Iowa during Summer and Fall 2024 (Figure 2e and Table S11), we measured a first liter lead concentration of 4.96 ppb from the KI kiosk in Baxter Springs, KS, and the lead concentration only decreased to 1.54 ppb in the fifth liter sample. Follow-up retesting of this KI kiosk in Baxter Springs, KS, found the lead concentration in the first liter reached a concerning 19.1 ppb, a serious health risk at nearly twice the US EPA action level, and the lead concentration remained at 1.67 ppb in the fifth liter. Zinc was also unexpectedly high at 864 ppb but decreased to 36 ppb after being flushed (Figure 2f). Outside of Iowa, we also found low (<1 ppb) but detectable levels of lead in water purchased from kiosks in Joplin, MO (TI), Miami, OK (TI), and Rogers, AR (Polar Station; PS). Although these lead levels are below all existing advisory levels, including the AAP’s 1 ppb threshold, the presence of copper and zinc provides evidence that plumbing components within the kiosks are corroding. Therefore, the potential exists for higher lead levels from plumbing components in response to changing water quality and/or extended periods of low use.

Collectively, over the course of monitoring 20 kiosks in Iowa and surrounding states, lead was detected (>0.05 μg/L, our method detection limit) in 15 kiosks; 5 kiosks dispensed water with lead >1 μg/L (the AAP recommendation), 2 kiosks dispensed water with lead >5 μg/L (the FDA allowable level for bottled water), and 1 kiosk dispensed water with lead >10 μg/L (US EPA Action Level) (see Table S12). For sampling locations with paired kiosk and tap water samples (Figure S6), median lead levels were higher in the first and fifth liter of kiosk water samples (0.30 and 0.18 μg/L, respectively) compared to the median values observed in the first and fifth liter of tap water samples (0.12 and 0.12 μg/L, respectively). As shown in Figure S6, the distribution of first and fifth liter kiosk water samples also tended to produce more elevated lead levels than those observed in the first and fifth liter tap water samples.

These data suggest that kiosks from select manufacturers, particularly HP, TI, and KI, represent an overlooked, long-term source of lead in drinking water. For example, for the HP kiosk in Muscatine, IA, we collected 15 water samples over the course of our study; lead was detected (>0.05 μg/L) in all 15 samples, lead was >1 μg/L in 4 samples, and it was >5 μg/L in 1 sample. Similarly, the KI kiosk in Baxter Springs, KS, was sampled 4 times over the course of our study; lead was detected and >1 μg/L in all samples and >10 μg/L in 1 sample. Because of the consistent detection of lead in select kiosks over extended time periods, these brands of kiosks may present a lead exposure risk, particularly for repeat customers.

### Mechanism of Lead Release into Kiosk Water

In all instances of lead in kiosk water >1 μg/L, we observed good agreement between dissolved lead and total lead measurements (Table S13), consistent with lead being primarily present in the dissolved rather than particulate form. Moreover, for HP kiosks in Iowa using RO treatment, copper and zinc were highly correlated with lead (Figure 3a), consistent with a common source or sources in kiosk plumbing. Use of RO treatment by kiosks appears to promote metal dissolution, consistent with expectations from large-scale water systems that use RO for advanced treatment and must stabilize the RO-treated water through pH adjustment or remineralization to avoid corrosion problems during distribution.^21,22^ In addition, RO treatment removed total phosphorus, which is added for corrosion control by most of the Iowa CWS with kiosks in their service area (Table S2), to below or very near the detection limit, at all sites (see Tables S9–S11). Thus, we suspect that the near-complete removal of alkalinity, hardness, and phosphate during RO treatment leaves metal distribution plumbing downstream of RO treatment vulnerable to corrosion and at-risk for lead leaching into water. This would explain why lead levels in kiosk water were often above those detected in corresponding tap water samples, despite kiosks sourcing their water from that same tap water.

**Figure 3:**
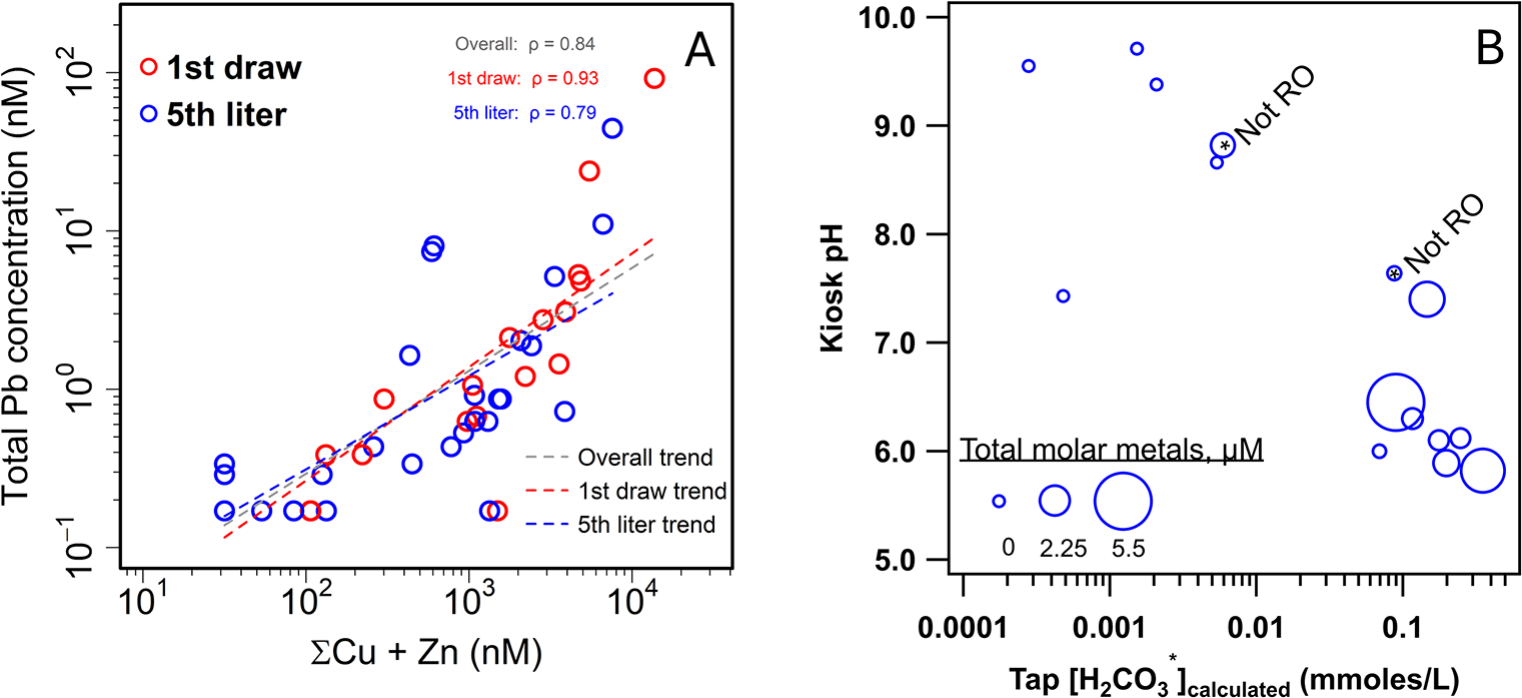
(A) Log–log plot of total lead concentration plotted against the sum of total copper and zinc (in nM) for 1st liter (red) kiosk samples (Spearman correlation ρ = 0.93, *p* < 0.001), 5th liter (blue) kiosk samples (Spearman correlation ρ = 0.79, *p* < 0.001), and both 1st liter and 5th liter samples (overall trend; purple dashed line, ρ = 0.84, *p* < 0.001). Note that the dashed line indicates the log–log trend and not Spearman’s ranked correlation slope. (B) Field pH of purchased kiosk water as a function of the sum of carbonic acid (H_2_CO_3_) and dissolved CO_2_ concentration (or H_2_CO_3_*) present in the corresponding tap water used as the kiosk source. Marker size represents the total molar sum of Cu, Zn, and Pb for the sample ranging from 0 to 5.5 μmol/L.

The amount of lead in kiosk water samples generally increased with lower pH values. From the analysis of paired tap and kiosk samples, we observed a clear bimodal distribution of kiosk water pH, with a cluster of kiosks with pH values near 8.5 and another cluster of kiosk water samples experiencing acidification after treatment with pH values of 6.5 or lower. The total metal content (on a molar basis) in kiosk samples was generally greatest for those samples at pH 6.5 or lower, which experienced acidification during RO treatment from the breakthrough of uncharged CO_2_ species [H_2_CO_3_* = CO_2_(aq) + H_2_CO_3_] (Figure 3b). Based on carbonate speciation in tap water samples and the relationship in Figure 3, we would anticipate that any tap water with pH ≤ 8.0 and alkalinity ≥ 50 mg/L as CaCO_3_ (≥0.02 mM H_2_CO_3_*) is likely to experience a ∼1.0 pH unit decrease through RO treatment at a kiosk. These conditions are most likely to generate lead in dispensed kiosk water.

In fact, statistical analysis revealed a strong positive correlation between the total molar sum concentration of Cu, Zn, and Pb (e.g., [Cu] + [Zn] + [Pb] in μmol/L) in first liter samples and predicted tap water H_2_CO_3_* concentration based on pH and alkalinity measurements (Spearman ρ = 0.77, *p-value* = 6.96 × 10^–4^). A moderately strong negative correlation between the molar sum of these 3 metals in the first liter samples and pH was also observed, which is consistent with pH-induced metal corrosion (Spearman ρ = −0.58, *p-value* = 8.73 × 10^–3^). Similarly strong correlations with pH and H_2_CO_3_* concentration were observed for the sum of Cu, Zn, and Pb in fifth liter samples and for these metals across all samples (1st and fifth liters) (Table S14), as well as for correlation analysis conducted with only Pb concentrations (Table S15).

### Presence of Lead in Purchased Ice from Kiosks

Lead contamination can also affect the ice purchased at kiosks. Of the 22 ice samples we collected over the course of our study, lead was detected (>0.05 μg/L) in 6 samples purchased from kiosks in Davenport, IA (HP), Baxter Springs, KS (KI), Joplin, MO (TI), St. Louis, MO (HP), Neosho, MO (TI), and Muscatine, IA (KI) (Table S16). Ice purchased from a TI kiosk in Neosho, MO, contained 10.6 ppb of lead, above the US EPA Action Level. The source of this lead is unclear because lead was below our method detection limit (0.05 ppb) in the water from this kiosk, and the kiosk did not appear to be employing RO treatment. This likely indicates another source in the ice production system that is susceptible to lead release, even without the corrosive conditions of RO-treated water. Our chemical analysis also suggests that treatment of water and ice as well as the resulting quality of each product can vary even at a single kiosk location.

### Stakeholder Engagement to Address Lead in Kiosk Water

Upon our initial discovery of lead in kiosk water, we shared our findings with both the Iowa Department of Natural Resources and US EPA Region 7 at the conclusion of our Summer 2024 sampling campaign. Through these conversations, it was clear that current policy regarding kiosks produces a regulatory gray zone: neither bureaucratic agency holds primary responsibility for water kiosks in Iowa, limiting their possible responses such that no additional action was taken. Next, we engaged local public health officials in every Iowa county with a kiosk. These discussions centered on alerting public health officials to our findings and providing them with the necessary information to answer questions from concerned kiosk customers. We also engaged drinking water utilities with kiosks in their service area and presented our findings at the Iowa American Water Works Association (IA AWWA) meeting to raise awareness among drinking water providers in the state.

During the revision of this article, with peer review providing more certainty of our findings, we engaged kiosk owners and manufacturers. We found local kiosk owners and suppliers to be receptive to our findings and eager to work with us toward a resolution. This afforded our team the opportunity to inspect kiosks from different manufacturers and use a portable X-ray Fluorescence (XRF) detector to inspect for potential lead-containing plumbing components. In a KI kiosk (Figures S7 and S8), we found one metal component downstream of the RO treatment that we suspect is nickel-plated brass, and XRF (Table S17) revealed that it contained 1.32% (w/w) of lead, although the abundance of lead along the wetted surface is unknown. In an HP kiosk, we found extensive use of brass fittings to connect PEX pipe and controls within the kiosk, including fittings downstream of RO in which XRF detected up to 0.17% (w/w) of lead.

In discussions with representatives of Kooler Ice, they shared that the part in question is a water meter that is compliant with NSF/ANSI 61 lead-free certification and the US EPA’s lead-free definition under the SDWA.^39^ SDWA §1417 establishes “lead free” as “not more than a weighted average of 0.25% lead calculated across the wetted surfaces of a pipe, pipe fitting, plumbing fitting, and fixture and 0.2% lead for solder and flux.” All of the brass fittings observed in the HP kiosk also meet his definition. Nevertheless, Pieper et al.^40^ found that plumbing components meeting this lead-free definition could still leach appreciable amounts of lead, especially under low pH and low alkalinity conditions and when such materials are used in consecutive connections. Thus, we conclude that the presence of lead in kiosk water is likely the result of manufacturers using lead-free plumbing components after RO, unaware that the small amounts of allowable lead under the SDWA can still be problematic upon exposure to low pH and low alkalinity waters that can be produced through RO treatment.

Manufacturers also told us that their machines were certified under the National Automatic Merchandising Association (NAMA)’s Machine Evaluation program. NAMA’s Machine Evaluation Program is a voluntary industry standard that conforms with the FDA’s Food Code. NAMA-certified machines are reevaluated annually to ensure ongoing compliance.^41^ The FDA Food Code limits the use of lead in utensils and food contact surfaces,^42^ but it is unclear whether it regulates lead in water vending machine equipment to the same degree as the SDWA’s requirement of lead-free components in drinking water systems.

### Implications and Opportunities for Improved Oversight

We have discovered widespread instances of lead release into water and ice purchased at kiosks manufactured by Kooler Ice, Highland Pure Water & Ice, and Twice the Ice. For some kiosks, lead release persists over time and can result in levels that exceed recommendations for vulnerable groups such as pregnant women and infants. The benefits posed by the ability of kiosks with RO treatment to remove PFAS from drinking water may not offset the risks they create by releasing lead into their product. Because we observed lead release into purchased water across different states and kiosk manufacturers, we contend that this problem likely presents a risk to consumers nationwide. For example, Twice the Ice and Highland Pure Water & Ice kiosks are produced by the same manufacturer and are prevalent throughout the country (see Figure 1), suggesting these problems may be widespread. Likewise, Kooler Ice claims over 1000 machines in the United States, Bahamas, Canada, and Australia.

Our findings demonstrate that kiosks’ marketing claims do not always reflect the product they deliver. In some cases, consumers pay a premium for kiosk water that is not significantly different from or even of lower quality than local tap water. Our results align with studies examining kiosks in other parts of the country,^10,12,19^ raising further concern about the water dispensed by kiosks nationwide. Given prior studies’ findings in underserved areas^9,10^ and kiosks’ location in areas with nonwhite, low socioeconomic status populations,^1^ historically marginalized individuals may be more likely to pay for expensive water that may not meet bottled water or tap water safety standards. These communities are already more likely to experience tap water problems,^43^ compounding the difficulties facing these groups.

Replacement of metal plumbing components in kiosks, especially those downstream of RO treatment, with materials (e.g., plastic and stainless steel) more compatible with RO-treated water should eliminate the issue of lead in the dispensed water. Kiosk manufacturers should alert individual owners to this problem and assist owners in replacing problematic parts. Unless lead-containing components are immediately replaced, kiosks at risk of corrosion may represent a long-term source of lead exposure through dispensed water for repeat customers.

Notably, this work highlights the shortcomings and unintended consequences arising from the current definition of “lead free” under the SDWA; lead-free components still allow for a percentage of lead that can be problematic when exposed to certain water quality types. Without a change of this definition to ensure that “lead-free” plumbing components are truly free of lead, there needs to be improved awareness about the problems that may arise from using certain types of metal plumbing components downstream of RO treatment for not only commercial kiosks but also all point-of-use and point-of-entry treatment scenarios (e.g., under-counter and whole-home RO systems).

A related issue is the need to reconsider NSF/ANSI testing protocols used to evaluate lead leaching from plumbing materials, especially test solutions meant to simulate more aggressive waters at low pH. In their work with leaded brass, Dudi et al.^44^ noted that the use of excess orthophosphate (77 mg/L as P) to buffer pH 5 test solutions resulted in markedly less lead leaching than pH 5 water free of orthophosphate, which is widely used as a corrosion inhibitor. As a result, they ultimately concluded that the testing protocols in NSF/ANSI 61, Section 8, were not highly protective of public health. We provide herein another example in which low pH, high purity water generated by RO treatment is extremely corrosive to lead in brass such that NSF/ANSI certification does not sufficiently protect from lead release.

Further, policy should also ensure that water dispensed by kiosks routinely meets SDWA standards and matches expectations from manufacturers’ marketing claims. The NAMA certification that kiosks in our study hold is a voluntary standard; not all water vending machines are “NAMA Listed.” One justification for kiosk’s light regulation is their classification as consecutive treatment systems, meaning kiosks are required to source their water from a public water supply. However, our results demonstrate that kiosks can deliver lower quality water than nearby tap water. We suggest that kiosks should be consistently regulated as nontransient noncommunity water systems under the SDWA. This classification would expand required kiosk testing to include lead and copper, while also more accurately reflecting repeated customer usage.^32^ Regulators could further limit lead in kiosk water to less than 5 ppb, as is the case for bottled water regulated by the FDA.

Policy can also increase transparency for customers. All kiosks should be required to have clear signage indicating the treatment they employ; in fact, for communities with PFAS contamination in their tap water, signage clearly indicating RO treatment, along with routine kiosk maintenance, is necessary to ensure that PFAS is being effectively removed. Kiosks should also post information about their source water, owner information, and the date of last service in multiple languages. In general, kiosks should be required to communicate water quality information with their customers so that customers can make informed decisions about the risks posed by possible microbial contamination, PFAS, and lead exposure. For example, it is unclear how kiosks respond to or alert the public about their own or source water quality problems.

We emphasize that our study was limited to a few types of kiosk manufacturers; we did not include all types of water vending machines. Realizing that consumers who rely on kiosks may want to seek out safer alternatives, we conducted a limited sampling of water vending machines located in local grocery stores in Iowa (“Natural Pure” Drinking Water in HyVee grocery stores). All samples from these in-store vending options were consistent with RO-treated water with lead below 0.05 μg/L. Also, over the duration of the current study, we conducted limited, convenience sampling in California of water vending machine brands Primo and Watermill Express. In these tests, water quality was consistent with RO treatment with lead below 0.05 μg/L in all cases. These results demonstrate that the problems observed with some kiosk companies herein are likely avoidable with proper construction and design as well as more rigorous regulatory oversight. Left unchecked, ambiguously regulated kiosks can risk environmental, ethical, and public health problems.

## Supplementary Material



## Notes

Additional details on materials, methods, results, and discussion (PDF)
