# Supplementary material for: Water Quality of U.S. Drinking Water Kiosks: Lead Release from “Lead-free” Plumbing after Reverse Osmosis Treatment
Source: Environ Sci Technol. 2026 Feb 11;60(7):5259–69. doi: 10.1021/acs.est.5c10647 (PMC12947684; doi:10.1021/acs.est.5c10647)
Supplement: Supplementary file 1 [file es5c10647_si_001.pdf]

*Supporting Information for*

**Water Quality of U.S. Drinking Water Kiosks: Lead Release from "Lead Free" Plumbing after Reverse Osmosis Treatment**

Samantha Zuhlke* (Samantha-zuhlke@uiowa.edu),[1] Drew E. Latta,[2, 3] Kate Beeman,[1] Amukta Gantalamohini,[1] James Kacer,[3] Grace Koch,[1] Danielle Land,[3,4,5] Abby McKeone,[1] Casie A. Meyer,[3] Matthew R. Nagorzanski,[3,6] Abdul H. Quraishi,[3] LilliAnna Scott,[1] Hanseob Shin,[3,6] Martin A. St. Clair,[2,3] Darrin A. Thompson,[3] David M. Cwiertny* (david-cwiertny@uiowa.edu)[2,3,4,7]

Prepared for *Environmental Science & Technology*

January 28, 2026

8 Figures, 17 Tables, 50 Pages

[1] School of Planning and Public Affairs, University of Iowa; Iowa City, IA, USA.
[2] IIHR-Hydroscience and Engineering, Department of Civil & Environmental Engineering; University of Iowa, Iowa City, IA, USA.
[3] Center for Health Effects of Environmental Contamination (CHEEC), University of Iowa; Iowa City, IA, USA.
[4] Department of Civil & Environmental Engineering, University of Iowa; Iowa City, IA, USA.
[5] C.S. Mott Department of Public Health, Michigan State University; Flint, MI, USA.
[6] State Hygienic Laboratory of Iowa, University of Iowa; Coralville, IA, USA.
[7] Department of Chemistry, University of Iowa; Iowa City, IA, USA.

## Supplementary Materials & Methods

**Sampling Details**. The Winter 2023 campaign collected samples at 10 kiosks across central and eastern Iowa, representing all known free-standing kiosks in Iowa at the time of sample collection. In Summer 2024, all 10 of the original kiosk locations were resampled, and samples were also collected at three additional kiosks (located in Washington, Muscatine, and Burlington) that were identified in Iowa after the Winter 2023 sampling event. In Summer of 2024, samples also were collected from nearby out-of-state locations in Missouri, Kansas, and Illinois. A final round of sampling in Fall 2024 (across September to December, 2024) was used for follow-up sampling of high priority kiosk locations (e.g., the Highland Pure kiosk in Muscatine, IA), a newly discovered Kooler Ice kiosk also in Muscatine, IA, as well as newly identified kiosk locations in Oklahoma and Arkansas. Collection of high priority samples at select locations also continued beyond Fall of 2024 and into Spring 2025. Across all sampling events, all kiosks were sampled twice, with the exception of those located in Burlington, IA; Moline, IL; Joplin, MO; St. Louis, MO; Rogers, AR, and Miami, OK. **Table S1** provides all details of kiosk sampling across the study period including kiosk location, the presence and contents of treatment signage, when and what types of samples were collected for analyses, and the location from where the comparison tap water sample was collected.

*Winter 2023 sampling*. In Winter 2023, samples were collected in the following order upon purchasing and dispensing of water from the kiosk: 4 liters of water for microbial analysis, 250 mL for metals (placing this sampling within the $5^{th}$ liter of dispensed water), 250 mL for major ions (e.g., chloride, nitrate) and general water quality indicators (e.g., pH and alkalinity), and then duplicate 250 mL bottles for PFAS analysis. For comparison, paired tap water samples were collected at a publicly accessible tap adjacent to each kiosk. Kiosk locations in Iowa and

details of the community water systems used as source water for kiosks are provided in **Table S2**. The sequence and volumes of water collected at all public taps were identical to those used for kiosks.

For PFAS samples, all water collection methods followed requirements from EPA Method 533 (*1*). Briefly, sampling kits included one 250 mL HDPE bottle (Environmental Sampling Supply) filled with organic free water (OFW; MilliQ IQ 7010) and four 250 mL HDPE sample bottles prefilled with 250 mg of ammonium acetate preservative (Environmental Sampling Supply). Once on site the bottle containing the OFW was poured into one of the HDPE sample bottles containing preservative to serve as a field reagent blank. The tap or kiosk water source was then used to fill the three remaining HDPE sample bottles.

After collecting all water samples, filled bottles were placed in an insulated cooler containing an ice pack(s) and transported back to the laboratory for analysis. Once samples arrived back to the laboratory, they were stored in a refrigerator at 4 °C until analysis. Samples for microbial analysis were processed within 24 hours, and samples for PFAS analysis were processed within the required holding time (28 days) for EPA Method 533.

*Summer and Fall 2024 sampling.* In Summer and Fall 2024, because of a primary focus on lead, sample collection at both kiosks and nearby taps followed $1^{st}$ and $5^{th}$ liter collection requirements from the Lead and Copper Rule Improvements. Additional samples for ionic water quality (250 mL) and PFAS (duplicate 250 mL with field blank) were then collected after the $5^{th}$ liter following approaches used in Winter 2023, although PFAS samples were only collected during the Summer 2024 sampling. Samples for microbial analysis were not collected in Summer and Fall 2024.

*Temporal sampling of HP Kiosk in Muscatine.* Temporal sample collection was conducted at one kiosk location, the HP kiosk in Muscatine, Iowa. This location was selected for its high level of lead in the 1st liter of dispensed water during initial sampling in Winter 2023. For temporal sampling, water was periodically purchased and collected from the kiosk over a ~24 h period between 10:30 pm on September 17, 2024, and 6:00 pm on September 18, 2024. For temporal sampling, each of the first 5-liters of water dispensed from the kiosk was collected for analysis in 1-liter HDPE bottles.

*Sampling of ice purchased from kiosks.* For sampling of ice, a 5 or 10 lbs. bag of ice was purchased from the kiosk. The ice was then transferred using a plastic cup into a 1-liter HDPE bottle, the same type of bottled used to collect water for lead analysis. The 1-liter bottle was filled completely with ice that was then allowed to melt, producing typically ~300 mL of water that was subsequently analyzed via ICP-MS for lead and other metals as described below.

**Field Measurements.** Most water samples were analyzed immediately upon collection for temperature, pH, and conductivity, and samples from Winter 2023 were also analyzed for free and total chlorine upon collection. Measurements of temperature, pH and conductivity were made using a Hach Pocket Pro+ Multi 1 Tester, which was calibrated within 24 h of use in the field. Measurements of free and total chlorine were conducted with a Hach DR300 Pocket Colorimeter, along with commercially available reagents through Hach for the use of the US EPA DPD method for free and total chlorine (Methods 8021 and 8167) (*2*, *3*).

**Field Blanks for Metals analysis.** For metals analysis field blanks, 1 liter of metals-free water was transferred into a standard sample collection bottle while at the kiosk. Field blanks

were analyzed for metal concentrations using Inductively Coupled Plasma Mass Spectrometry (ICP-MS) as described below. In all instances, the analytical results of field blank samples were all below method detection limits for target analytes.

**Microbial Analysis.** For enumeration of heterotrophic bacteria, coliforms/*E. coli*, and *Enterococci*, a 100 mL of kiosk and tap water was removed from the 4 L obtained for microbial analysis and used for heterotrophic plate count (HPC), Colilert, and Enterolert test kits (IDEXX Laboratories, Inc., Westbrook, ME, USA). Of the remaining water sample, 3 L was filtered through 0.45 µm pore size membrane filters (PALL, Ann Arbor, MI, United States) for DNA extraction, and the DNA was subsequently extracted from the membrane using a DNeasy PowerWater Kit (Qiagen; Germantown, MD) following the manufacturer's instructions. DNA concentrations were determined using NanoDrop One (Thermo Fisher Scientific). For microbiome analysis, **s**equencing of the hypervariable region, V3-V4, of the bacterial 16S rDNA was run as previously described (*4*). Raw sequence fastq files from the samples were processed, using the DADA2 pipeline (*5*). In brief, primers were initially removed, and sequences were truncated based on a Phred quality score threshold of 25, followed by denoising to correct base calling inaccuracies. Paired reads were then merged, and chimeras removed, resulting in the generation of amplicon sequence variants (ASVs) (*6*).Taxonomic classification of these ASVs was performed using the Silva database (Version 138.1, released March 2021). Fastq-formatted sequence data has been deposited at NCBI under accession number PRJNA1151119, with accession numbers: SAMN43303211, SAMN43303212, SAMN43303213, SAMN43303214, SAMN43303215, SAMN43303216, SAMN43303217, SAMN43303218, SAMN43303219,

SAMN43303220, SAMN43303221, SAMN43303222, SAMN43303223, SAMN43303224, SAMN43303225, SAMN43303220, SAMN43303220.

**PFAS Analysis.** Paired tap water and kiosk water samples were analyzed for PFAS chemicals using a slightly modified analytical method based on EPA Method 533 (*1*). This method targeted 25 different PFAS analytes (**Table S3**). Before concentration via solid phase extraction, the samples were weighed and then spiked with an isotope dilution analogue, or IDA. The IDA was made by combining and diluting commercially available isotopically labeled PFAS analytes (Wellington Laboratories). The samples were then concentrated using solid phase extraction (Promochom SPE-03 Gen4). Empty sample bottles were dried and weighed to determine sample volume. The extracts were dried to completion using a turbovap (Biotage TurboVap LV), then reconstituted with 1 mL of 80:20 Methanol:OFW. The reconstituted extracts were spiked with an internal performance standard, IPS, that was created by combining and diluting commercially available isotopically labeled PFAS analytes (Wellington Laboratories). The extracts were then aliquoted into LC vials for analysis (SureSTART™ 2 mL Polypropylene Screw Top Microvials for < 2 mL Samples, Level 1 Everyday Analysis, Thermofisher). Sample analysis was conducted using liquid chromatography (Agilent 1290) tandem mass spectrometry (SciEx 6500+). Quality control was assured by analyzing a laboratory reagent blank and lab fortified blank spiked with a native PFAS analyte solution (Phenova) every batch of twenty samples. A matrix spike and matrix spike duplicate are also analyzed used a random sample every batch.

**Metals Analysis.** Samples for dissolved metals were prepared prior to digestion by filtering 10 mL of sample past a 13 mm diameter 0.45 µm polypropylene membrane syringe tip filter (Tisch Scientific) into 14 mL ICP-MS tubes. Filtered aliquots were acidified with 0.4 mL of 1:1 v/v trace metal grade nitric acid (Fisher Scientific) to DI water. Samples for total metals analysis were prepared by adding 20 mL aliquots of trace metals grade nitric acid to 1 liter of sample stored at 4 °C. In addition, 5 mL of 40% w/v hydroxylamine hydrochloride solution (to achieve 0.2 wt% hydroxylamine). The solution of 40 % w/v hydroxylamine prepared from deionized water and solid hydroxylamine hydrochloride (Thermo Scientific, 99+%). Samples were digested in an oven for 24 hours at 50 °C. Samples were then cooled and aliquoted to 14 mL ICP-MS tubes. Metals analyses were performed by inductively coupled plasma mass spectrometry (ICP-MS, Agilent 7900, Agilent Technologies) following a modified EPA method 200.8.1 The method was modified to include major elements Na, Mg, K, Ca, and Fe which were measured in He collision gas mode to extend the high-concentration dynamic range and to remove 56ArO+ (m/z = 56) interference on 56Fe. Similar QA/QC procedures (QC solutions, blanks, fortified blanks, and spiked samples) were followed with the major elements as for the EPA method 200.8 metals.

**Ion Analysis.** Ion chromatography (IC) analysis of anions utilized a Metrohm 930 Compact IC Flex equipped with a Metrosep A Supp 19-100/4.0 column using a sodium carbonate /sodium hydrogen carbonate eluant or a Dionex ICS 6000 with Dionex IonPac AS19-4 µm column and hydroxide eluant. A Metrohm 919 IC Autosampler Plus with in-line ultrafiltration was used for sampling. Instrument blanks were run daily, and did not indicate significant levels of contamination. Check standards were analyzed after every ten samples on

the ion chromatograph. Limits of detection (in mg/L) were 0.0025 for fluoride, 0.0084 for chloride, 0.0037 for nitrate (as N) and 0.0068 for sulfate. Limits of quantification (in mg/L) were 0.008 for fluoride, 0.026 for chloride, 0.011 for nitrate (as N) and 0.021 for sulfate.

**Portable X-ray fluorescence investigation.** We were allowed to inspect both Kooler Ice and Highland Pure Water and Ice Kiosks in Muscatine, IA, by their owners and maintenance staff. During our inspection we were able to conduct portable X-ray fluorescence spectroscopy (Thermo Scientific Niton XL3t portable XRF) on metal fittings within the kiosks. XRF spectra were collected using the metal alloys mode of the XRF and collected for 30 to 35 seconds. Alloy identification was given by the software routines included with the instrument.

## Supplemental Results and Discussion

**Temperature and Residual Chlorine of Kiosk Water**. There was no consistency as to whether water delivered from a kiosk was chilled (see **Table 1** of Main Text). Temperature was highly variable across kiosk samples, spanning from chilled water (a low temperature of 13.6 °C in Davenport, IA during our Summer 2024 sampling) to ambient temperature that was comparable to, if not warmer than, corresponding tap water (e.g., kiosk water from Fairfield, IA, was 33.6 °C during our Summer 2024 sampling). In some instances, we also noted that the temperature of the kiosk water sample would decrease while dispensing. We attribute this to the ambient warming of stagnant water nearest the kiosk dispenser.

For some RO-treated kiosk water in Iowa, measurements often revealed little to no chlorine residual (**Table S4**). In Winter 2023, residual levels of total chlorine were below the limit of detection in water dispensed from kiosks in Clinton, Muscatine and Fort Madison and less than the corresponding value in tap water from kiosks in Des Moines, one location in Davenport, Ottumwa, Mt. Pleasant and Fairfield. Lower residual chlorine in kiosk water is likely due to the expected break down and removal of residual disinfectant present in the tap water source during RO treatment.

**Microbial water quality**. Although the loss of disinfectant residual during RO treatment raises concerns about microbial growth within the kiosk plumbing, we did not find evidence of significant microbial contamination in HP kiosks in Iowa. Enumeration of heterotrophic plate counts (HPC) demonstrated that 3 of the 10 tap water samples from Iowa had 20 MPNs/100 mL, whereas no HPC was measurable in any of the kiosk samples. Further, total coliforms/*E. coli* and

*Enterococci* were not detected in any tap or kiosk water sample (**Table S5**). DNA extraction and sequencing generally revealed lower microbial abundance in kiosk water relative to tap water. Although we were able to extract sufficient DNA for microbiome analysis from all tap water samples, only 6 kiosk samples produced sufficient quantities for analysis.

In the kiosk water samples, the dominant phyla were Proteobacteria, Actinobacteria, and Firmicutes (**Figure S1**). Proteobacteria exhibited the highest relative abundance, ranging from 22.03% to 61.54%. Actinobacteria also showed considerable abundance, with percentages ranging from 11.54% to 39.13%, followed by Firmicutes (4.95% – 25.42%). In the tap water samples, Proteobacteria was also the most dominant phyla, ranging from 26.97% to 63.98%, followed by Actinobacteria (2.80% – 26.61%). Firmicutes showed variable abundance (0.62% – 26.25%) in tap water.

Proteobacteria is a well-known phylum predominantly found in various environmental settings including the natural environment and drinking water. In particular, Betaproteobacteria, which are common in drinking water, can include opportunistic pathogens with antibiotic resistance and virulence factors (*7*). In addition, they can form biofilms, allowing them to persist during disinfection and in distribution systems even with residual disinfectant, and they have evolved mechanisms to adapt to various environmental niches that may help lead them to human hosts (*8*).

Our microbiome analysis also allowed us to explore for genetic evidence of opportunistic pathogens, with four genera of opportunistic pathogens identified in select kiosk and tap water samples at the genus level (**Table S6**). *Mycobacterium* - spp. was detected in samples from five kiosks (Des Moines, both in Davenport, Ottumwa and Fairfield; 0.19 - 1.96%) and all taps (0.37 - 3.75%). This genus is known for causing nontuberculous mycobacterial (NTM) infections,

which can affect the lungs and skin (*9*). Notably, higher prevalence was observed in tap water from Clinton (3.37%), Muscatine (2.41%), and Fort Madison (3.75%). *Legionella* spp., potentially responsible for a severe form of pneumonia (*10*), was identified only in tap water samples from Des Moines, both locations in Davenport, Bettendorf, Ottumwa and Fairfield, ranging from 0.17% to 1.25%. *Acinetobacter* spp., which can cause a range of infections including respiratory, urinary tract, and bloodstream infections (*11*), was found in all kiosk samples with sufficient DNA for microbiome analysis (i.e., Des Moines, both locations in Davenport, Ottumwa, Mt. Pleasant and Fairfield; 0.12% – 5.08%) and tap water samples from both locations in Davenport, Bettendorf, Muscatine, Ottumwa, Mt. Pleasant, Fort Madison and Fairfield (0.09% - 4.68%). *Pseudomonas* spp., particularly *Pseudomonas aeruginosa*, which causes infections in the blood, lungs, and other parts of the body (*12*) was detected in kiosks in Des Moines, Ottumwa, Mt. Pleasant and Fairfield (0.44% - 3.84%), and tap water samples from Clinton, one location in Davenport (Davenport – 2), Ottumwa, Mt. Pleasant and Fairfield (0.29% - 3.12%).

Although these observations suggest the possible occurrence of opportunistic pathogens, we cannot say with confidence that any of these specific species are present because microbiome analysis only identified bacterial organisms up to the genus level. These findings, along with those other studies of water from vending machines (*10,11*) and growing awareness of opportunistic pathogens in drinking water distribution systems through metagenomic approaches (*13*), suggest the need for more monitoring of drinking water focused on emerging pathogenic risks.

The microbial composition based on amplicon sequence variants (ASVs) of tap and kiosk water samples were compared by using Bray-Curtis dissimilarity index (**Figure S2**). The

kiosk and tap water samples showed significant difference in microbial composition (PERMANOVA, non-linear $R^2 = 0.996$, stress = 0.108141, $p < 0.05$), although four tap water samples were clustered with the kiosk group. Those four tap water samples correspond to the water systems that source groundwater, whereas all other tap water samples sourced from surface water showed more significant microbial composition variability. Close association of paired kiosk and tap water samples from Mt. Pleasant and Fort Madison suggests that the microbial composition of both the tap and kiosk water are likely shaped by the cities' source water and/or distribution system. Microbial compositions in both kiosk locations in Davenport and the kiosk in Ottumwa are particularly interesting because they deviate significantly from their corresponding tap water composition, all of which is sourced from surface waters. After treatment by reverse osmosis (RO), the microbial composition of the kiosk water closely associates with those found in other tap water samples sourced from groundwater. We speculate this may simply reflect the limited abundance and diversity of microbes likely to be encountered in groundwater (relative to surface water) and after RO treatment, which can effectively remove many microorganisms.

**PFAS removal during kiosk treatment**. From Winter 2023 sampling in Iowa (**Figure S3a and 3b, Table S7**), our analysis found detectable levels [above 1 part-per-trillion (ppt) or 1 nanogram per liter] of total PFAS chemicals in tap water samples from Des Moines (5.1 ppt total PFAS), Davenport (two tap locations at 26.3 and 28.6 ppt), Bettendorf (27.4 ppt), Muscatine (77.9 ppt), and Ottumwa (12.1 ppt), whereas tap water from Clinton, Mt. Pleasant, Fort Madison, and Fairfield had no detectable PFAS. Although most of the PFAS in Muscatine tap water was present as PFBA (51.0 ppt), it also contained PFOA (3.8 ppt) and PFOS (5.4 ppt) at levels close

to or above their recently proposed Maximum Contaminant Levels (MCLs).  All water systems with detectable PFAS rely on surface water sources including the Mississippi River (e.g., Davenport and Bettendorf; see **Figure S4**) except for Muscatine, which sources from the Muscatine Island Alluvial Aquifer that is partially recharged by the Mississippi River. Notably, all systems without detectable PFAS (i.e., Clinton, Mt. Pleasant, Fort Madison, and Fairfield) rely on groundwater sources.

We observed very little variation in total PFAS levels at locations in Iowa resampled during Summer 2024 (**Figure S3c and S3d and Table S8**). Burlington, IA, which sources primarily from the Mississippi River along with three alluvial wells, and Washington, IA, which relies on groundwater, were also sampled in Summer 2024. Although PFAS was not detected in Washington tap water, 44.8 ppt of total PFAS, including PFOA and PFOS, was measured in Burlington tap water. Outside of Iowa, low-level PFAS was also detected in tap water samples collected in Joplin, MO (4.7 ppt), Baxter Springs, KS (16.1 ppt), and Moline, IL (22.8 ppt), with PFAS present as a mixture of both short and long-chain species, including PFOA and PFOS.

Analysis of paired water samples from kiosks suggests that their use of RO treatment is generally effective at removing most, if not all, PFAS species. Through two rounds of sampling in Iowa, we only observed a small amount of PFBA (1.5 ppt) in the RO-treated water from the HP kiosk in Muscatine in Winter 2023. This corresponds to 98% removal of PFBA during RO treatment (relative to the tap water sample) and removal of all other PFAS species detected in Muscatine tap water to levels below detection. All other RO-treated kiosk samples in Iowa were free of PFAS in Winter 2023 and Summer of 2024, including the Muscatine HP kiosk sample from Summer of 2024.

In Summer of 2024, there were two observations of PFAS in samples from kiosks not using RO treatment. In Joplin, MO, we detected both PFBS (1.1 ppt) and PFPeA (1.7 ppt) in water from a TI kiosk using microfiltration, activated carbon filtration, cation exchange and UV disinfection. Although a small amount of PFOS (1.8 ng/L) was detected in Joplin tap water sample, PFOS was not detected in the kiosk sample. Likely, the activated carbon filter, which typically performs better for the removal of longer chain PFAS species (*14*), is responsible for the removal of PFOS and the breakthrough of shorter chain PFBS and PFPeA. Several PFAS species were also detected in water from the KI kiosk in Muscatine, IA, but the total was notably lower than typically observed in Muscatine tap water. We suspect, therefore, the KI kiosk may be using activated carbon treatment, which would not impact dissolved ions (consistent with results in **Table 1** of the main text) but may help reduce the total amount of PFAS present in the kiosk water.

**Additional Corrosion-based Metals Results.** *1st and 5th liter results from all kiosks.* Metal concentrations determined via ICP-MS in the 5th liter of Winter 2023 sampling and 1st and 5th liters for Summer 2024 and Fall 2024 sampling are shown in **Tables S9-S11**. Metal concentrations from the repeat sampling of the KI kiosk in Muscatine, IA in March 2025 is also included in Table S10. These tables include common corrosion-based metals (copper, zinc, tin and lead) and total phosphorous, which is typically added to water as part of corrosion control efforts. Generally, total phosphorous was consistently higher in corresponding tap water samples when compared to kiosk water samples, suggesting that the RO treatment provided by kiosks removes corrosion control protection. For sampling when 1st and 5th liter samples were collected

from kiosks (Summer 2024 and Fall 2024; Tables S9 and S10), we also note that levels of lead, copper and zinc were generally lower in the 5$^{th}$ liter compared to the 1$^{st}$ liter.

*Temporal sampling of HP Kiosk in Muscatine.* For temporal sampling (**Figure S5**), lead, copper and zinc were measured in each of the first 5 liters of water dispensed from the kiosk over a 24-hour period. Consistent with water stagnation increasing lead levels, we observed the highest levels of lead (~1.0 ppb), zinc and copper in the 1$^{st}$ liter sample collected in the early morning (5:30 AM) of September 18, 2024, a level that was higher than that measured in the sample collected at 10:30 PM the prior evening (September 17, 2024). Lead, copper and zinc concentrations subsequently decreased in the 2$^{nd}$, 3$^{rd}$, 4$^{th}$ and 5$^{th}$ liter of water in the early morning sample, the expected concentration profile for flushing of corrosion-based metals located in premise plumbing. Notably, samples collected at midday and the evening of September 18, 2024, showed a more constant concentration profile for all metals across the 1$^{st}$ to 5$^{th}$ liter, as anticipated during periods of greater kiosk use (i.e., without opportunity for stagnation to replenish the dissolved metal concentrations).

*Analysis of ice*. Our analysis also reveals that the quality of ice was somewhat variable and not always representative of the water sold at the kiosk. The ice at five locations had the same or higher cation concentrations (**Table S14**) than their associated kiosk water, whereas the remainder of ice sample exhibited lower concentrations of cations relative to the kiosk water.

**Kiosk inspection for lead-containing components**. Upon inspection, the KI kiosk appeared to employ a series of pre-treatment filters prior to RO. Only one of these filters was clearly labeled as “Sediment”, and we were unable to clearly identify the other types of pre-treatment used. We suspect that the other pre-treatment filter is for dechlorination, as is common

in such decentralized applications of RO. The pretreated water is then fed to the RO system, after which it is moved to storage in the water reservoir.

The water reservoir was located below a large reservoir for ice (although the ice production system was not clearly visible). The owner did not know the dimensions or volume of the water reservoir, but it appeared to be roughly 4 to 5 feet wide and likely only about three feet deep because it was located in the rear half of the kiosk (the dimensions on a Kooler Ice kiosk are reported by the manufacturer to be 7 ft wide, 7.5 feet deep, and 11 ft tall). Prior to dispensing, the water was passed through one final filter, which was not clearly labeled but we suspect is an activated carbon filter (we note that the treatment processed used by KI kiosks are not listed on the outside of the kiosk).

We were able to identify two metal fittings in the water treatment and delivery system, only one of which was after RO treatment. This metal part was situated after the presumed activated carbon filter and in line to the dispenser. The metal piece appeared to be a flow meter or control valve, although we were only able to inspect the bottom of the fitting visually. With use of a portable X-ray Fluorescence (XRF) meter, we detected measurable amounts of lead (0.56% at the connector leaving the carbon filter and 1.32% when measured at the circular portion of the fitting which we presume is a flow meter). The other elements detected appear to suggest it is nickel-plated brass (**Table S15**). Thus, for KI kiosks, we suspect that the lead is likely entering into the water as it passes through this lead-containing, metal plumbing component after RO treatment and immediately prior to dispensing.

Inspection of the Highland Pure Water and Ice kiosk in Muscatine, Iowa (manufactured by Twice the Ice), revealed the use of multiple consecutive brass fittings used to connect PEX plastic pipe with most PEX-to-PEX elbows, tees, and PEX to threaded NPT fittings on plumbing

controls being made of brass. General flow of water was from the back of the unit where the filters, carbon, RO and reservoir were located to the front of the unit, necessitating the use of multiple fittings to route PEX piping through the kiosk. XRF analysis of select brass fittings revealed a mix of brass compositions with one tee having 0.17% Pb and another having 0.029% Pb. An elbow analyzed contained 0.05% lead. The outside of the water-contact portion of a pressure-reducing valve contained 0.14% Pb. We note that all of the fittings analyzed in the Highland Pure kiosk meet the definition of lead-free as defined in SDWA at less that 0.25% lead.

Our inspection and XRF analysis provide clear evidence of a source of lead in legal components that come into contact with RO-treated water. Low water use and consecutive use of brass within the Highland Pure kiosk may explain the presence of over 9 ppb of lead in the 5$^{th}$ liter of the Highland Pure kiosk in Muscatine, Iowa, in our Winter 2023 sampling. Based on the design of the Highland Pure kiosk in Muscatine, Iowa, the combined feed system from the reservoir to both the water and ice maker that contained brass fittings could also explain the lead in ice dispensed from select kiosks. Our general findings agree with a YouTube video that includes footage from inside a Twice the Ice kiosk unit without RO treatment.

# Supplementary Tables

**Table S1.** Details of kiosk sampling across Winter 2023 (W23), Summer 2024 (Su24), Fall 2024 (F24) and Spring 2025 (Sp25) sampling campaigns. An "X" indicates a sample was collected for the corresponding analysis.

| | | | | | | Analyses | | | | | | | | | |
|---|---|---|---|---|---|---|---|---|---|---|---|---|---|---|---|
| Kiosk Location | Address | County | Brand | Treatment signage | Sampling Dates | Metals | PFAS | Biological | Major Ions | Water Quality (pH) | Chlorine | Field Probe | Paired Tap Water Sample | Tap Water Sampling Location (distance from kiosk) | Ice Collected |
| Des Moines | 3310 SE 14th St Des Moines, IA 50320 | Polk | HP | Branded as "Purified by Reverse Osmosis" | W23 | 5th Liter | X | X | X | X | X | X | X | Walgreens, 3140 SE 14th St, Des Moines, IA 50320 (0.2 mile) | |
| | | | | | Su24 | 1st & 5th Liter | | | X | X | | X | X | Buffalo Wild Wings, 3311 SE 14th St, Des Moines, IA 50320 (0.1 mile) | X |
| Clinton | 340 5th Ave S, Clinton, IA 52732 | Clinton | HP | Micron filtration, Micron filtration, activated carbon filtration, reverse osmosis, ion exchange, UV light cleansing | W23 | 5th Liter | X | X | X | X | X | X | X | Walgreens, 806 S 4th St, Clinton, IA 52732 (0.5 mile) | |
| | | | | | Su24 | 1st & 5th Liter | | | X | X | | X | X | Walgreens, 806 S 4th St, Clinton, IA 52732 (0.5 mile) | X |
| Davenport-HP1 | 3833 N Division St, Davenport, IA 52806 | Scott | HP | Micron filtration, Micron filtration, activated carbon filtration, reverse osmosis, ion exchange, UV light cleansing | W23 | 5th Liter | X | X | X | X | X | X | X | Walgreens, 1720 W Kimberly Rd, Davenport, IA 52806 (0.1 mile) | |
| | | | | | Su24 | 1st & 5th Liter | | | X | X | | X | X | Walgreens,1720 W. Kimberly Rd, Davenport, IA 52806 (0.1 mile) | X |
| Davenport-HP2 | 2402 E 53rd St, Davenport, IA 52807 | Scott | HP | Micron filtration, Micron filtration, activated | W23 | 5th Liter | X | X | X | X | X | X | X | Jimmy Johns, 3537 Middle Rd, Bettendorf, IA 52722 (4.8 mile) | |

| | | | | | | | | | | | | | | | |
|---|---|---|---|---|---|---|---|---|---|---|---|---|---|---|---|
| | | | | carbon filtration, reverse osmosis, ion exchange, UV light cleansing | Su24 | 1st & 5th Liter | | | X | X | | X | X | Jimmy Johns, 2406 E. 53rd St., Davenport, IA 52807 (250 ft) | X |
| Bettendorf | 2925 18th St, Bettendorf, IA 52722 | Scott | HP | Micron filtration, Micron filtration, activated carbon filtration, reverse osmosis, ion exchange, UV light cleansing | W23 | 5th Liter | X | X | X | X | X | X | X | Public Library, 2950 Learning Campus Dr, Bettendorf, IA 52722 (0.2 mile) | |
| | | | | | Su24 | 1st & 5th Liter | | | X | X | | X | X | CVS, 2002 Spruce Hills Dr, Bettendorf, IA 52722 (0.3 mile) | X |
| Muscatine-HP | 901 Cypress St, Muscatine, IA 52761 | Muscatine | HP | Micron filtration, Micron filtration, activated carbon filtration, reverse osmosis, ion exchange, UV light cleansing | W23 | 5th Liter | X | X | X | X | X | X | X | Hy-Vee, 510 E 6th St, Muscatine, IA 52761 (0.6 mile) | |
| | | | | | Su24 | 1st & 5th Liter | | | X | X | | | X | Hy-Vee, 510 E 6th St, Muscatine, IA 52761 (0.6 mile) | X |
| | | | | | F24 | 1st, 2nd, 3rd, 4th, & 5th Liter | | | X | X | | | | | |
| | | | | | W24 | 1st & 5th Liter | | | X | X | | | | | |
| Muscatine-KI | 714 Grandview Ave, Muscatine, IA 52761 | Muscatine | KI | Branded as “Filtered Water” | F24 | 1st & 5th Liter | | | X | X | | | X | | |
| | | | | | Sp25 | 1st & 5th Liter | | | X | X | | | X | | X |
| Ottumwa | 819 Albia Rd, Ottumwa, IA 52501 | Wapello | HP | Micron filtration, Micron filtration, activated carbon filtration, reverse osmosis, ion exchange, UV light cleansing | W23 | 5th Liter | X | X | X | X | X | X | X | BP Gas Station, 720 Richmond Ave, Ottumwa, IA 52501 (0.1 mile) | X |
| | | | | | Su24 | 1st & 5th Liter | | | X | X | | X | X | BP Gas Station, 720 Richmond Ave, Ottumwa, IA 52501 (0.1 mile) | |
| Mt. Pleasant | 304 W Washington St, Mt | Henry | HP | Micron filtration, Micron filtration, | W23 | 5th Liter | X | X | X | X | X | X | X | Casey’s, 400 W Washington St, Mt Pleasant, IA 52641 | X |

| | | | | | | | | | | | | | | | |
|---|---|---|---|---|---|---|---|---|---|---|---|---|---|---|---|
| | Pleasant, IA 52641 | | | activated carbon filtration, reverse osmosis, ion exchange, UV light cleansing | | | | | | | | | | (243 ft) | |
| | | | | | Su24 | 1st & 5th Liter | | | X | X | | X | X | Casey's, 400 W Washington St, Mt Pleasant, IA 52641 (243 ft) | |
| Fort Maddison | 1702 Ave H, Fort Madison, IA 52627 | Lee | HP | Micron filtration, Micron filtration, activated carbon filtration, reverse osmosis, ion exchange, UV light cleansing | W23 | 5th Liter | X | X | X | X | X | X | X | BP Gas Station, 1737 Ave H, Fort Madison, IA 52627 (394 ft) | X |
| | | | | | Su24 | 1st & 5th Liter | | | X | X | | X | X | BP Gas Station, 1737 Ave H, Fort Madison, IA 52627 (394 ft) | |
| Fairfield | 2 N 9th St, Fairfield, IA 52556 | Jefferson | HP | Micron filtration, Micron filtration, activated carbon filtration, reverse osmosis, ion exchange, UV light cleansing | W23 | 5th Liter | X | X | X | X | X | X | X | Hy-Vee Gas Station, 1310 W Burlington Ave, Fairfield, IA 52556 (0.3 mile) | X |
| | | | | | Su24 | 1st & 5th Liter | | | X | X | | X | X | Hy-Vee Gas Station, 1310 W Burlington Ave, Fairfield, IA 52556 (0.3 mile) | |
| Washington | 400 S. B Ave, Washington, IA 52353 | Washington | HP | Micron filtration, Micron filtration, activated carbon filtration, reverse osmosis, ion exchange, UV light cleansing | Su24 | 1st & 5th Liter | | | X | X | | X | X | Phillips 66 Gas Station, 100 E. Madison St., Washington, IA 52353 (0.6 mile) | X |
| Burlington | 1014 Division St., Burlington, IA 52601 | Des Moines | HP | Micron filtration, Micron filtration, activated carbon filtration, reverse osmosis, ion exchange, UV light cleansing | Su24 | 1st & 5th Liter | | | X | X | | X | X | Walgreens, 201 S Central Ave, Burlington, IA 52601 (0.1 mile) | X |
| Moline | 4100 12th Ave, Moline, IL 61265 | Rock Island | HP | Micron filtration, Micron filtration, activated | Su24 | 1st & 5th Liter | | | X | X | | X | X | Fast and Fresh Gas Station, 4228 Avenue of the Cities, Moline, IL 61265 | X |

| | | | | | | | | | | | | | | | |
|---|---|---|---|---|---|---|---|---|---|---|---|---|---|---|---|
| | | | | carbon filtration, reverse osmosis, ion exchange, UV light cleansing | | | | | | | | | | (1.0 mile) | |
| Baxter Springs | 2204 Military Ave, Baxter Springs, KS 66713 | Cherokee | KI | Branded as “Filtered Water” | Su24 | 1st & 5th Liter | | | X | X | | X | X | Casey’s Gas Station, 2403 Military Ave, Baxter Springs, KS 66713 (0.2 miles) | X |
| | | | | | F24 | 1st & 5th Liter | | | X | X | | | | | X |
| Joplin | 702 S Maiden Ln, Joplin, MO 64801 | | TI | Micron Filtration, Activated Carbon Filtration, Ion Exchange, Ultraviolet Light Cleansing | Su24 | 1st & 5th Liter | | | X | X | | X | | McDonald’s 1531 W 7th St, Joplin, MO 64801 (0.1 mile) | X |
| St. Louis | 3799 St Monica Ln, St Ann, MO 63074 | Clayton | HP | Micron Filtration, Activated Carbon Filtration, Ion Exchange, Micron Filtration, Reverse Osmosis, Ultraviolet Light Cleansing | Su24 | 1st & 5th Liter | | | X | X | | | X | 10330 Natural Bridge Rd, St. Louis, MO 63134 (3.2 mile) | X |
| Neosho | 909 W Harmony St, Neosho, MO 64850 | Newton | TI | Marketed as “Fresh filtered water and ice” | Su24 | 1st & 5th Liter | | | X | X | | X | | | X |
| | | | | | F24 | 1st & 5th Liter | | | X | X | | | X | Briar and Thistle Restaurant, 107 E Main St, Neosho, MO 64850 (1.1 mile) | X |
| Rogers | 14991 E Hwy 12, Rogers, AR 72756 | Benton | PS | Marketed as “Purified Ice and Water” | F24 | 1st & 5th Liter | | | X | X | | | X | Casey’s Gas Station 514 N 2nd St, Rogers, AR 72756 (3.6 miles) | X |
| Miami | 2020 N Main St, Miami, OK 74354 | Ottawa | TI | Micron filtration, Ion Exchange, Activated | F24 | 1st & 5th Liter | | | X | X | | | | | X |

| | | | | | | | | | | | |
|---|---|---|---|---|---|---|---|---|---|---|---|
| Carbon Filtration, Ultraviolet Light Cleansing | | | | | | | | | | | |

**Table S2.** HP Kiosk locations in Iowa and information about the community water systems from which HP kiosks source water. All information was obtained from respective state databases about public water systems that are publicly available through Iowa Department of Natural Resources.

| City | County | Kiosk Location | Community Water System | Source Water | Disinfectant | Corrosion Control | PWSID |
|---|---|---|---|---|---|---|---|
| Des Moines | Polk | 3310 SE 14th St. Des Moines, IA 50320 | Des Moines Water Works | SW | Chlorination | Alkalinity (>40 mg/L), pH (>8.2), phosphate (>0.25 mg/L) | IA7727031 |
| Clinton | Clinton | 340 5th Ave S, Clinton, IA 52732 | IA American Water – Clinton | GW | Chlorination | Polyphosphate† | IA2326048 |
| Davenport-HP1 | Scott | 3833 N Division St, Davenport, IA 52806 | IA American Water - Davenport | SW | Chlorination, Chloramination (DBP control) | Orthophosphate (0.9 to 1.4 mg/L), pH > 7.2 | IA8222001 |
| Davenport-HP2 | Scott | 2402 E 53rd St, Davenport, IA 52807 | IA American Water - Davenport | SW | Chlorination, Chloramination (DBP control) | Orthophosphate (0.9 to 1.4 mg/L), pH (> 7.2) | IA8222001 |
| Bettendorf | Scott | 2925 18th St, Bettendorf, IA 52722 | IA American Water - Davenport | SW | Chlorination Chloramination (DBP control) | Orthophosphate (0.9 to 1.4 mg/L), pH (> 7.2) | IA8222001 |
| Muscatine | Muscatine | 901 Cypress St, Muscatine, IA 52761 | Muscatine Power & Water | GW | Chlorination | Ortho-polyphosphate blend† | IA7048001 |
| Ottumwa | Wapello | 819 Albia Rd B, Ottumwa, IA 52501 | Ottumwa Water Works | SW | Chlorination | Alkalinity, polyphosphate† | IA9083012 |
| Mt. Pleasant | Henry | 304 W Washington St, Mt Pleasant, IA 52641 | Mt. Pleasant Municipal Utilities | GW | Chlorination | Alkalinity, Ortho-polyphosphate blend (partial system use)† | IA4453016 |
| Fort Madison | Lee | 1702 Ave H, Fort Madison, IA 52627 | Fort Madison Municipal Water Works | GW | Chlorination | Alkalinity, pH, Zinc phosphate† | IA5625062 |
| Fairfield | Jefferson | 2 N 9th St, Fairfield, IA 52556 | Fairfield Water Supply | GW | Chlorination, Chloramination | Phosphate, polyphosphate | IA5131033 |
| Washington | Washington | 400 S. B Ave, Washington, IA 52353 | Washington Water Department | GW | Chlorination | pH, zinc orthophosphate† | IA9271068 |
| Burlington | Des Moines | 1014 Division St., Burlington, IA 52601 | Burlington Municipal Water Works | SW | Chlorination | Polyphosphate† | IA2909053 |

†Iowa DNR does not always provide detailed information on the type of phosphate corrosion inhibitor used, particularly for systems serving fewer than the 50,000 people (per SDW A). The operating permit of medium-sized and smaller systems does not specify type and concentration of CCT chemical – and typically only requires dosing information (lbs./day, etc.). CCT information can be extracted from narratives in Sanitary Survey documents, however, these documents are only publicly available to 2020. In several cases polyphospha te is stated to be added for sequestration of metals rather than specifically as a corrosion control inhibitor.

**Table S3.** Full list of PFAS analytes used in EPA Method 533 as used in this study

| Analytes | Abbreviation | CASRN | Chain Length |
|---|---|---|---|
| Perfluorobutanoic acid | PFBA | 375-22-4 | 4 |
| Perfluorobutanesulfonic acid | PFBS | 375-73-5 | 4 |
| Perfluoro(2-ethoxyethane)sulfonic acid | PFEESA | 113507-82-7 | 4 |
| Perfluoro-3-methoxypropanoic acid | PFMPA | 377-73-1 | 4 |
| Nonafluoro-3,6-dioxaheptanoic acid | NFDHA | 151772-58-6 | 5 |
| Perfluoro-4-methoxybutanoic acid | PFMBA | 863090-89-5 | 5 |
| Perfluoropentanoic acid | PFPeA | 2706-90-3 | 5 |
| Perfluoropentanesulfonic acid | PFPeS | 2706-91-4 | 5 |
| 1H,1H, 2H, 2H-Perfluorohexane sulfonic acid | 4:2FTS | 757124-72-4 | 6 |
| Hexafluoropropylene oxide dimer acid | HFPO-DA | 13252-13-6 | 6 |
| Perfluorohexanoic acid | PFHxA | 307-24-4 | 6 |
| Perfluorohexanesulfonic acid | PFHxS | 355-46-4 | 6 |
| 4,8-Dioxa-3H-perfluorononanoic acid | ADONA | 919005-14-4 | 7 |
| Perfluoroheptanoic acid | PFHpA | 375-85-9 | 7 |
| Perfluoroheptanesulfonic acid | PFHpS | 375-92-8 | 7 |
| 1H,1H, 2H, 2H-Perfluorooctane sulfonic acid | 6:2FTS | 27619-97-2 | 8 |
| 9-Chlorohexadecafluoro-3-oxanonane-1-sulfonic acid | 9Cl-PF3ONS | 756426-58-1 | 8 |
| Perfluorooctanoic acid | PFOA | 335-67-1 | 8 |
| Perfluorooctanesulfonic acid | PFOS | 1763-23-1 | 8 |
| Perfluorononanoic acid | PFNA | 375-95-1 | 9 |
| 11-Chloroeicosafluoro-3-oxaundecane-1-sulfonic acid | 11Cl-PF3OUdS | 763051-92-9 | 10 |
| 1H,1H, 2H, 2H-Perfluorodecane sulfonic acid | 8:2FTS | 39108-34-4 | 10 |
| Perfluorodecanoic acid | PFDA | 335-76-2 | 10 |
| Perfluoroundecanoic acid | PFUnA | 2058-94-8 | 11 |
| Perfluorododecanoic acid | PFDoA | 307-55-1 | 12 |

**Table S4.** Levels of free and total chlorine measured in purchased HP kiosk water samples in Iowa and corresponding tap water samples collected nearby. Data were collected during the Winter 2023 sampling campaign.

| | Kiosk | | Tap | |
|---|---|---|---|---|
| **City** | **Free Cl** | **Total Cl** | **Free Cl** | **Total Cl** |
| Des Moines | 0.04 | 0.12 | 0.6 | 0.54 |
| Clinton | 0 | 0 | 0.38 | 0.44 |
| Davenport-HP1 | 0.07 | 0.4 | 0.02 | 0.07 |
| Davenport-HP2 | 0.02 | 0.07 | 0.24 | > 2.0* |
| Bettendorf | 0.13 | 0.23 | 0.01 | 0.05 |
| Muscatine | 0 | 0 | 0.47 | 0.59 |
| Ottumwa | 0.17 | 0.158 | 0.14 | >2.0* |
| Mt. Pleasant | 0 | 0.07 | 1.39 | 1.35 |
| Fort Madison | 0 | 0 | 1.38 | 1.51 |
| Fairfield | 0.2 | 0.24 | 0.28 | > 2.0* |

**Value was above the range of detection for the colorimetric analysis*

**Table S5.** Most probable numbers (MPNs) per 100 mL of sample for total heterotrophic bacteria, total coliforms and *E. coli*, and total Enterococci from HP kiosk and tap water samples collected in Iowa in Winter 2023.

| Location | Type | Most Probable Number (MPN)/100mL | | | |
|---|---|---|---|---|---|
| | | Total Heterotrophic Bacteria | Total coliforms | Total *E. coli* | Total *Enterococci* |
| Des Moines | Tap | 0 | 0 | 0 | 0 |
| | Kiosk | 0 | 0 | 0 | 0 |
| Clinton | Tap | 0 | 0 | 0 | 0 |
| | Kiosk | 0 | 0 | 0 | 0 |
| Davenport-HP1 | Tap | 20 | 0 | 0 | 0 |
| | Kiosk | 0 | 0 | 0 | 0 |
| Davenport-HP2 | Tap | 20 | 0 | 0 | 0 |
| | Kiosk | 0 | 0 | 0 | 0 |
| Bettendorf | Tap | 20 | 0 | 0 | 0 |
| | Kiosk | 0 | 0 | 0 | 0 |
| Muscatine | Tap | 0 | 0 | 0 | 0 |
| | Kiosk | 0 | 0 | 0 | 0 |
| Ottumwa | Tap | 0 | 0 | 0 | 0 |
| | Kiosk | 0 | 0 | 0 | 0 |
| Mt. Pleasant | Tap | 0 | 0 | 0 | 0 |
| | Kiosk | 0 | 0 | 0 | 0 |
| Fort Madison | Tap | 0 | 0 | 0 | 0 |
| | Kiosk | 0 | 0 | 0 | 0 |
| Fairfield | Tap | 0 | 0 | 0 | 0 |
| | Kiosk | 0 | 0 | 0 | 0 |

**Table S6**. Relative abundance of genus identified in HP kiosk and tap water samples in Winter 2023 based on 16S rDNA amplicon sequencing data with analysis of amplicon Sequence Variants (ASVs). Genera with a relative abundance below 0.1% were grouped as “minor” category. Locations: 1 – Des Moines; 2 – Clinton; 3 – Davenport HP1; 4 – Davenport HP2; 5 – Bettendorf; 6 – Muscatine; 7 – Ottumwa; 8 – Mt. Pleasant; 9 – Fort Madison; 10 – Fairfield.

| **Genus** | **Relative abundance (%)** | | | | | | | | | | | | | | | |
|---|---|---|---|---|---|---|---|---|---|---|---|---|---|---|---|---|
| | **K1** | **K3** | **K4** | **K7** | **K8** | **K10** | **T1** | **T2** | **T3** | **T4** | **T5** | **T6** | **T7** | **T8** | **T9** | **T10** |
| OD1_genus_incertae_sedis | 0 | 1.69 | 1.44 | 0 | 0 | 0 | 0 | 0.56 | 2.05 | 4.2 | 0.12 | 0 | 0 | 0 | 0 | 2.62 |
| *Methylobacterium* | 2.5 | 0 | 1.44 | 1.96 | 0 | 2.1 | 0 | 0.56 | 0.18 | 2.54 | 1.49 | 0 | 0.56 | 0 | 3.75 | 0.18 |
| *Sphingomonas* | 2.12 | 0 | 2.89 | 2.94 | 3.84 | 5.26 | 1.24 | 0 | 1.02 | 0.97 | 0.62 | 2.41 | 0.68 | 1.56 | 1.25 | 0.42 |
| *Planctomyces* | 0.25 | 0 | 0 | 0 | 0 | 0 | 0.17 | 0.56 | 2.05 | 0.97 | 3.74 | 0.8 | 0.45 | 0 | 0 | 0.42 |
| *Mycobacterium* | 0.19 | 1.69 | 1.44 | 1.96 | 0 | 1.05 | 1.06 | 3.37 | 0.37 | 1.56 | 1.12 | 2.41 | 1.02 | 1.56 | 3.75 | 0.48 |
| *Nitrospira* | 0.25 | 0 | 0 | 0 | 0 | 0 | 0.17 | 1.68 | 1.96 | 0.39 | 1.12 | 0 | 0.34 | 1.56 | 0 | 1.15 |
| *Blastomonas* | 0.7 | 0 | 0 | 0 | 0 | 0 | 5.31 | 0 | 0 | 0 | 0.62 | 0 | 0.9 | 0 | 0 | 0 |
| *Legionella* | 0 | 0 | 0 | 0 | 0 | 0 | 0.17 | 0 | 1.02 | 0.97 | 0.74 | 0 | 1.25 | 0 | 0 | 0.91 |
| *Acinetobacter* | 0.12 | 5.08 | 1.44 | 1.96 | 3.84 | 2.1 | 0 | 0 | 0.28 | 0.09 | 0.37 | 2.41 | 1.81 | 4.68 | 1.25 | 0.3 |
| *Sphingopyxis* | 0.12 | 0 | 1.44 | 0 | 0 | 0 | 3.01 | 0 | 0.18 | 0.19 | 1.12 | 0 | 0.45 | 0 | 2.5 | 0.42 |
| *Deinococcus* | 2.5 | 0 | 0 | 0 | 0 | 1.05 | 0.17 | 0 | 0 | 0.09 | 0 | 0 | 0 | 0 | 2.5 | 0.06 |
| *TM7_genus_incertae_sedis* | 1.34 | 1.69 | 0 | 0 | 0 | 0 | 0 | 0.56 | 0 | 0.09 | 0 | 0 | 0 | 0 | 0 | 1.22 |
| *Gemmatimonas* | 0.83 | 0 | 0 | 0 | 0 | 0 | 0 | 0.56 | 0.65 | 0.09 | 1.99 | 0 | 0.22 | 0 | 0 | 0.12 |
| *Sediminibacterium* | 0.06 | 0 | 0 | 0 | 0 | 3.15 | 0.17 | 0.56 | 1.68 | 0.68 | 0.12 | 0 | 0.79 | 0 | 0 | 0.06 |
| *Brevundimonas* | 0.19 | 0 | 0 | 0 | 0 | 0 | 1.77 | 0.56 | 0.37 | 0.09 | 0.12 | 0 | 1.36 | 0 | 0 | 0.24 |
| *Pseudoxanthomonas* | 0.19 | 0 | 0 | 0 | 0 | 0 | 4.25 | 0 | 0.18 | 0 | 0.12 | 0 | 0.56 | 0 | 0 | 0.06 |
| *Flavobacterium* | 0.83 | 0 | 0 | 0 | 0 | 1.05 | 0 | 1.12 | 0.37 | 0.78 | 0.12 | 0 | 0.22 | 1.56 | 1.25 | 0.12 |
| *Hyphomicrobium* | 0.19 | 0 | 0 | 0 | 0 | 0 | 1.06 | 0.56 | 0.84 | 0.39 | 0.37 | 0.8 | 0.56 | 0 | 0 | 0.18 |
| *Singulisphaera* | 1.09 | 1.69 | 0 | 0 | 0 | 0 | 0.53 | 0 | 0.18 | 0.09 | 0.37 | 0 | 0.45 | 0 | 0 | 0.24 |
| *Corynebacterium* | 0.44 | 3.38 | 5.79 | 2.94 | 0 | 2.1 | 0.35 | 0 | 0 | 0.09 | 0.12 | 4.03 | 0.22 | 3.12 | 1.25 | 0.12 |
| *Propionibacterium* | 0.12 | 8.47 | 4.34 | 2.94 | 3.84 | 2.1 | 0.35 | 0.56 | 0.09 | 0.29 | 0.12 | 1.61 | 0.34 | 3.12 | 2.5 | 0.06 |
| *Vampirovibrio* | 0 | 0 | 0 | 0 | 0 | 1.05 | 0.7 | 1.12 | 0.46 | 0.29 | 2.12 | 0 | 0.11 | 0 | 0 | 0.06 |
| *Hymenobacter* | 2.05 | 0 | 1.44 | 0 | 0 | 0 | 0 | 0 | 0 | 0 | 0 | 0 | 0 | 0 | 0 | 0 |
| *Bdellovibrio* | 0.12 | 0 | 0 | 0 | 0 | 0 | 0 | 0 | 0.93 | 0.48 | 0 | 0 | 0 | 0 | 0 | 0.91 |
| *Pseudomonas* | 0.44 | 0 | 0 | 1.96 | 3.84 | 3.15 | 0 | 1.12 | 0 | 0.29 | 0 | 0 | 0.68 | 3.12 | 0 | 0.36 |
| Unclassified | 53.98 | 32.2 | 44.92 | 37.25 | 50 | 36.84 | 63.82 | 63.48 | 71.3 | 70.64 | 70.03 | 47.58 | 64.43 | 39.06 | 41.25 | 71.32 |
| **Minor (< 0.1%)** | 29.24 | 44.06 | 33.33 | 46.07 | 34.61 | 38.94 | 15.6 | 23.03 | 13.73 | 13.6 | 13.23 | 37.9 | 22.5 | 40.62 | 38.75 | 17.87 |

**Table S7.** Total and individual PFAS concentrations in paired tap and HP kiosk samples in Winter 2023. Numbers with a less than sign "<" denote samples below the method reporting limit. Analytes included in EPA Method 533 but not shown were below method reporting limit for all samples. All concentrations are presented in parts-per-trillion (ppt) or nanograms per liter.

| Winter 2023 | | PFAS | | | | | | | | | | |
|---|---|---|---|---|---|---|---|---|---|---|---|---|
| **Location** | **Sample** | **Total PFAS** | **PFOA** | **PFOS** | **PFNA** | **PFBS** | **HFPO-DA** | **PFHxS** | **PFBA** | **PFPeA** | **PFHxA** | **PFHpA** |
| Des Moines, IA | Tap | 5.1 | <1.0 | 1.2 | <1.0 | 1.2 | <1.0 | 1.2 | 1.6 | <1.0 | <1.0 | <1.0 |
| | Kiosk | <1.0 | <1.0 | <1.0 | <1.0 | <1.0 | <1.0 | <1.0 | <1.0 | <1.0 | <1.0 | <1.0 |
| Clinton, IA | Tap | <1.0 | <1.0 | <1.0 | <1.0 | <1.0 | <1.0 | <1.0 | <1.0 | <1.0 | <1.0 | <1.0 |
| | Kiosk | <1.0 | <1.0 | <1.0 | <1.0 | <1.0 | <1.0 | <1.0 | <1.0 | <1.0 | <1.0 | <1.0 |
| Davenport, IA-HP1 | Tap | 26.3 | 2.9 | 1.6 | <1.0 | 1.4 | <1.0 | <1.0 | 13.3 | 2.1 | 1.9 | <1.0 |
| | Kiosk | <1.0 | <1.0 | <1.0 | <1.0 | <1.0 | <1.0 | <1.0 | <1.0 | <1.0 | <1.0 | <1.0 |
| Davenport, IA-HP2 | Tap | 28.6 | 2.9 | 1.6 | <1.0 | 1.2 | <1.0 | <1.0 | 15.5 | 2.4 | 1.8 | <1.0 |
| | Kiosk | <1.0 | <1.0 | <1.0 | <1.0 | <1.0 | <1.0 | <1.0 | <1.0 | <1.0 | <1.0 | <1.0 |
| Bettendorf, IA | Tap | 27.4 | 3.0 | 1.9 | <1.0 | 1.3 | <1.0 | <1.0 | 15.1 | 2.5 | 1.9 | <1.0 |
| | Kiosk | <1.0 | <1.0 | <1.0 | <1.0 | <1.0 | <1.0 | <1.0 | <1.0 | <1.0 | <1.0 | <1.0 |
| Muscatine, IA | Tap | 77.9 | 3.8 | 5.4 | <1.0 | 4.3 | <1.0 | 1.3 | 51.0 | 6.7 | 5.4 | <1.0 |
| | Kiosk | 1.5 | <1.0 | <1.0 | <1.0 | <1.0 | <1.0 | <1.0 | 1.5 | <1.0 | <1.0 | <1.0 |
| Ottumwa, IA | Tap | 12.1 | 1.0 | 1.2 | <1.0 | 1.4 | <1.0 | <1.0 | 3.2 | 2.8 | 2.5 | <1.0 |
| | Kiosk | <1.0 | <1.0 | <1.0 | <1.0 | <1.0 | <1.0 | <1.0 | <1.0 | <1.0 | <1.0 | <1.0 |
| Mt Pleasant, IA | Tap | <1.0 | <1.0 | <1.0 | <1.0 | <1.0 | <1.0 | <1.0 | <1.0 | <1.0 | <1.0 | <1.0 |
| | Kiosk | <1.0 | <1.0 | <1.0 | <1.0 | <1.0 | <1.0 | <1.0 | <1.0 | <1.0 | <1.0 | <1.0 |
| Fort Madison, IA | Tap | <1.0 | <1.0 | <1.0 | <1.0 | <1.0 | <1.0 | <1.0 | <1.0 | <1.0 | <1.0 | <1.0 |
| | Kiosk | <1.0 | <1.0 | <1.0 | <1.0 | <1.0 | <1.0 | <1.0 | <1.0 | <1.0 | <1.0 | <1.0 |
| Fairfield, IA | Tap | <1.0 | <1.0 | <1.0 | <1.0 | <1.0 | <1.0 | <1.0 | <1.0 | <1.0 | <1.0 | <1.0 |
| | Kiosk | <1.0 | <1.0 | <1.0 | <1.0 | <1.0 | <1.0 | <1.0 | <1.0 | <1.0 | <1.0 | <1.0 |

**Table S8.** Total and individual PFAS concentrations in paired tap and kiosk samples in Summer 2024 and Fall 2024. Numbers with a less than sign "<" denote samples below the method reporting limit. Analytes included in EPA Method 533 but not shown below were below method reporting limit for all samples. All concentrations are presented in parts-per-trillion (ppt) or nanograms per liter.

| Summer 2024 | | PFAS | | | | | | | | | | |
|---|---|---|---|---|---|---|---|---|---|---|---|---|
| Location | Sample | Total PFAS | PFOA | PFOS | PFNA | PFBS | HFPO-DA | PFHxS | PFBA | PFPeA | PFHxA | PFHpA |
| Des Moines, IA | Tap | 10.9 | <1.0 | 2.4 | <1.0 | 1.6 | <1.0 | 2.7 | 2.0 | 1.0 | 1.1 | <1.0 |
| | Kiosk | <1.0 | <1.0 | <1.0 | <1.0 | <1.0 | <1.0 | <1.0 | <1.0 | <1.0 | <1.0 | <1.0 |
| Clinton, IA | Tap | <1.0 | <1.0 | <1.0 | <1.0 | <1.0 | <1.0 | <1.0 | <1.0 | <1.0 | <1.0 | <1.0 |
| | Kiosk | <1.0 | <1.0 | <1.0 | <1.0 | <1.0 | <1.0 | <1.0 | <1.0 | <1.0 | <1.0 | <1.0 |
| Davenport, IA-HP1 | Tap | 19.1 | 2.3 | 1.6 | <1.0 | 1.4 | <1.0 | <1.0 | 10.9 | 1.6 | 1.2 | <1.0 |
| | Kiosk | <1.0 | <1.0 | <1.0 | <1.0 | <1.0 | <1.0 | <1.0 | <1.0 | <1.0 | <1.0 | <1.0 |
| Davenport, IA-HP2 | Tap | 18.4 | 2.2 | 1.4 | <1.0 | 1.5 | <1.0 | <1.0 | 10.7 | 1.4 | 1.2 | <1.0 |
| | Kiosk | <1.0 | <1.0 | <1.0 | <1.0 | <1.0 | <1.0 | <1.0 | <1.0 | <1.0 | <1.0 | <1.0 |
| Bettendorf, IA | Tap | 18.4 | 2.4 | 1.3 | <1.0 | 1.4 | <1.0 | <1.0 | 10.7 | 1.4 | 1.2 | <1.0 |
| | Kiosk | <1.0 | <1.0 | <1.0 | <1.0 | <1.0 | <1.0 | <1.0 | <1.0 | <1.0 | <1.0 | <1.0 |
| Muscatine, IA | Tap | 74.2 | 3.8 | 5.6 | <1.0 | 5.1 | <1.0 | 1.2 | 49.3 | 5.0 | 4.3 | <1.0 |
| | Kiosk | 1.5 | <1.0 | <1.0 | <1.0 | <1.0 | <1.0 | <1.0 | 1.5 | <1.0 | <1.0 | <1.0 |
| Ottumwa, IA | Tap | 7.3 | <1.0 | 1.1 | <1.0 | <1.0 | <1.0 | <1.0 | 3.7 | 1.2 | 1.2 | <1.0 |
| | Kiosk | <1.0 | <1.0 | <1.0 | <1.0 | <1.0 | <1.0 | <1.0 | <1.0 | <1.0 | <1.0 | <1.0 |
| Mt Pleasant, IA | Tap | <1.0 | <1.0 | <1.0 | <1.0 | <1.0 | <1.0 | <1.0 | <1.0 | <1.0 | <1.0 | <1.0 |
| | Kiosk | <1.0 | <1.0 | <1.0 | <1.0 | <1.0 | <1.0 | <1.0 | <1.0 | <1.0 | <1.0 | <1.0 |
| Fort Madison, IA | Tap | <1.0 | <1.0 | <1.0 | <1.0 | <1.0 | <1.0 | <1.0 | <1.0 | <1.0 | <1.0 | <1.0 |
| | Kiosk | <1.0 | <1.0 | <1.0 | <1.0 | <1.0 | <1.0 | <1.0 | <1.0 | <1.0 | <1.0 | <1.0 |
| Fairfield, IA | Tap | <1.0 | <1.0 | <1.0 | <1.0 | <1.0 | <1.0 | <1.0 | <1.0 | <1.0 | <1.0 | <1.0 |
| | Kiosk | <1.0 | <1.0 | <1.0 | <1.0 | <1.0 | <1.0 | <1.0 | <1.0 | <1.0 | <1.0 | <1.0 |
| Washington, IA | Tap | <1.0 | <1.0 | <1.0 | <1.0 | <1.0 | <1.0 | <1.0 | <1.0 | <1.0 | <1.0 | <1.0 |
| | Kiosk | <1.0 | <1.0 | <1.0 | <1.0 | <1.0 | <1.0 | <1.0 | <1.0 | <1.0 | <1.0 | <1.0 |

| | | | | | | | | | | | | |
|---|---|---|---|---|---|---|---|---|---|---|---|---|
| Burlington, IA | Tap | 44.8 | 4.1 | 2.0 | <1.0 | 1.5 | <1.0 | 1.0 | 24.3 | 9.7 | 2.3 | <1.0 |
| | Kiosk | <1.0 | <1.0 | <1.0 | <1.0 | <1.0 | <1.0 | <1.0 | <1.0 | <1.0 | <1.0 | <1.0 |
| Moline, IL | Tap | 22.8 | 2.6 | 1.4 | <1.0 | 1.8 | <1.0 | <1.0 | 13.5 | 1.9 | 1.6 | <1.0 |
| | Kiosk | <1.0 | <1.0 | <1.0 | <1.0 | <1.0 | <1.0 | <1.0 | <1.0 | <1.0 | <1.0 | <1.0 |
| Baxter Springs, MO | Tap | 16.1 | 1.5 | 2.3 | <1.0 | 1.8 | <1.0 | <1.0 | 1.9 | 3.5 | 4.1 | 1 |
| | Kiosk | <1.0 | <1.0 | <1.0 | <1.0 | <1.0 | <1.0 | <1.0 | <1.0 | <1.0 | <1.0 | <1.0 |
| Joplin, MO | Tap | 4.7 | <1.0 | 1.8 | <1.0 | 1.2 | <1.0 | <1.0 | <1.0 | 1.7 | <1.0 | <1.0 |
| | Kiosk | 2.8 | <1.0 | <1.0 | <1.0 | 1.1 | <1.0 | <1.0 | <1.0 | 1.7 | <1.0 | <1.0 |

| Fall 2024 | | PFAS | | | | | | | | | | |
|---|---|---|---|---|---|---|---|---|---|---|---|---|
| **Location** | **Sample** | **Total PFAS** | **PFOA** | **PFOS** | **PFNA** | **PFBS** | **HFPO-DA** | **PFHxS** | **PFBA** | **PFPeA** | **PFHxA** | **PFHpA** |
| Muscatine, IA | Kiosk-HP | <1.0 | <1.0 | <1.0 | <1.0 | <1.0 | <1.0 | <1.0 | <1.0 | <1.0 | <1.0 | <1.0 |
| Muscatine, IA | Kiosk-KI | 36.0 | 1.7 | <1.0 | <1.0 | 2.6 | <1.0 | <1.0 | 26.0 | 3.2 | 2.5 | <1.0 |

**Table S9.** Plumbing-based metals and total phosphorus determined in paired tap and HP kiosk samples during the Winter 2023 sampling campaign. Numbers with a less than sign “<” denote samples below the method detection limit.

| Winter 2023 (5th Liter) | | | | | | |
|---|---|---|---|---|---|---|
| **Location** | **Sample** | **Copper** | **Zinc** | **Tin** | **Lead** | **Total P** |
| | | **µg/L** | **µg/L** | **µg/L** | **µg/L** | **µg/L** |
| Clinton, IA | Tap | 500 | 8.76 | <0.05 | 0.31 | 282 |
| | Kiosk | 38.2 | 31.5 | <0.05 | 0.13 | <7.98 |
| Mt. Pleasant, IA | Tap | 22.2 | 7.2 | <0.05 | 0.07 | 150 |
| | Kiosk | 50.9 | 83.6 | <0.05 | 0.42 | <7.98 |
| Ft. Madison, IA | Tap | 21.3 | 500 | <0.05 | 0.08 | 524 |
| | Kiosk | 17.9 | 9.88 | <0.05 | 0.34 | <7.98 |
| Muscatine, IA | Tap | 496 | 7.32 | 0.11 | 0.1 | 402 |
| | Kiosk – HP | 231 | 257 | <0.05 | 9.23 | 8.17 |
| Bettendorf, IA | Tap | 322 | 19.1 | 0.07 | 0.24 | 471 |
| | Kiosk | 125 | 90.1 | 0.09 | 1.07 | <7.98 |
| Davenport, IA – HP1 | Tap | 114 | 28.6 | 0.12 | 0.36 | 412 |
| | Kiosk | 49.3 | 34.6 | <0.05 | 0.13 | <7.98 |
| Davenport, IA – HP2 | Tap | 15.4 | 7.65 | <0.05 | <0.05 | 472 |
| | Kiosk | 228 | 199 | 0.08 | 2.29 | <7.98 |
| Ottumwa, IA | Tap | 27.1 | 3.98 | <0.05 | 0.06 | 206 |
| | Kiosk | <0.8 | <2.1 | <0.05 | 0.06 | <7.98 |
| Fairfield, IA | Tap | 15.3 | 31.5 | <0.05 | 0.14 | 436 |
| | Kiosk | <0.8 | <2.1 | <0.05 | <0.05 | <7.98 |
| Des Moines, IA | Tap | 11.3 | 3.42 | <0.05 | 0.09 | 146 |
| | Kiosk | <0.8 | <2.1 | <0.05 | 0.07 | <7.98 |

**Table S10.** Plumbing-based metals and total phosphorus determined in paired tap and kiosk samples during the Summer 2024 sampling campaign. Numbers with a less than sign "<" denote samples below the method detection limit.

| Summer 2024 | | 1st Liter | | | | | 5th Liter | | | | |
|---|---|---|---|---|---|---|---|---|---|---|---|
| Location | Sample | Copper | Zinc | Tin | Lead | Total P | Copper | Zinc | Tin | Lead | Total P |
| | | μg/L | μg/L | μg/L | μg/L | μg/L | μg/L | μg/L | μg/L | μg/L | μg/L |
| Clinton, IA | Tap | 552 | 7.86 | <0.05 | 0.17 | 394 | 303 | 6.39 | <0.05 | 0.2 | 415 |
| | Kiosk | 63 | 121 | <0.05 | 0.57 | 9.26 | 11.1 | 5.54 | <0.05 | 0.09 | <7.98 |
| Mt. Pleasant, IA | Tap | 19.1 | 6.74 | <0.05 | 0.05 | 110 | 14.1 | 2.76 | <0.05 | <0.05 | 96.9 |
| | Kiosk | 31.3 | 40.4 | <0.05 | 0.14 | <7.98 | 16 | 12.8 | <0.05 | 0.07 | <7.98 |
| Ft. Madison, IA | Tap | 29.7 | 379 | <0.05 | 0.07 | 348 | 24.1 | 387 | <0.05 | <0.05 | 360 |
| | Kiosk | 6.39 | 7.87 | <0.05 | 0.08 | <7.98 | 3.36 | 5.24 | <0.05 | <0.05 | <7.98 |
| Burlington, IA | Tap | 37.4 | 21.8 | 0.07 | 1.05 | 299 | 26 | 3.82 | <0.05 | 0.31 | 354 |
| | Kiosk | <0.8 | <2.1 | <0.05 | <0.05 | <7.98 | <0.8 | <2.1 | <0.05 | <0.05 | <7.98 |
| Muscatine, IA | Tap | 423 | 8.31 | <0.05 | <0.05 | 353 | 379 | 3.26 | <0.05 | <0.05 | 347 |
| | Kiosk – HP | 42.3 | 20.2 | <0.05 | 0.13 | <7.98 | 35.1 | 14.6 | 0.09 | 0.09 | <7.98 |
| Bettendorf, IA | Tap | 11.7 | 4.3 | <0.05 | 0.06 | 346 | 5.25 | <2.1 | <0.05 | <0.05 | 402 |
| | Kiosk | 52.7 | 14.5 | <0.05 | 0.22 | <7.98 | 46.4 | 12.7 | <0.05 | 0.11 | <7.98 |
| Davenport, IA - HP1 | Tap | 73.6 | 46.9 | <0.05 | 0.23 | 317 | 38 | 9.24 | <0.05 | 0.07 | 330 |
| | Kiosk | 149 | 102 | <0.05 | 0.64 | <7.98 | 67.5 | 29.7 | <0.05 | 0.18 | <7.98 |
| Davenport, IA - HP2 | Tap | 8.9 | 4.9 | <0.05 | <0.05 | 346 | 7.06 | <2.1 | <0.05 | <0.05 | 376 |
| | Kiosk | 75.7 | 38 | <0.05 | 0.44 | <7.98 | 72.8 | 28.1 | 0.09 | 0.18 | <7.98 |
| Ottumwa, IA | Tap | 44.6 | <2.1 | <0.05 | <0.05 | 136 | 25 | <2.1 | <0.05 | <0.05 | 131 |
| | Kiosk | <0.8 | <2.1 | <0.05 | <0.05 | <7.98 | <0.8 | <2.1 | <0.05 | <0.05 | <7.98 |
| Moline, IL | Tap | 43.6 | 29.9 | <0.05 | 0.09 | 43.4 | 30 | <2.1 | <0.05 | <0.05 | 38.4 |
| | Kiosk | <0.8 | <2.1 | <0.05 | <0.05 | <7.98 | <0.8 | <2.1 | <0.05 | <0.05 | <7.98 |
| Fairfield, IA | Tap | 39.9 | 38 | <0.05 | 0.06 | 373 | 19.7 | 34.5 | <0.05 | 0.07 | 361 |
| | Kiosk | <0.8 | <2.1 | <0.05 | <0.05 | <7.98 | <0.8 | <2.1 | <0.05 | <0.05 | <7.98 |
| Washington, IA | Tap | 1.01 | 218 | <0.05 | <0.05 | 295 | 1.17 | 236 | <0.05 | <0.05 | 305 |
| | Kiosk | 1.15 | 96.4 | <0.05 | <0.05 | 294 | 0.87 | 86.4 | <0.05 | <0.05 | 277 |
| Des Moines, IA | Tap | 9.16 | 46.3 | <0.05 | <0.05 | 151 | 6.86 | <2.1 | <0.05 | <0.05 | 147 |
| | Kiosk | <0.8 | <2.1 | <0.05 | <0.05 | <7.98 | <0.8 | <2.1 | <0.05 | <0.05 | <7.98 |
| Baxter Springs, KS | Tap | 18.5 | 7.03 | <0.05 | 0.24 | 15.7 | 12.4 | 3.37 | <0.05 | 0.18 | 17 |
| | Kiosk - KI | 26.7 | 330 | <0.05 | 4.96 | <7.98 | 14.4 | 23.7 | 0.07 | 1.54 | <7.98 |
| Joplin, MO | Tap | 96.8 | 8.09 | 0.12 | 0.12 | 43 | 44.5 | 4 | 0.93 | 0.12 | 40.9 |

| | | | | | | | | | | | |
|---|---|---|---|---|---|---|---|---|---|---|---|
| | Kiosk | 11.9 | 7.44 | <0.05 | 0.18 | 43.9 | 5.96 | 2.07 | <0.05 | 0.06 | 40 |
| St. Louis, MO | Tap | 5.46 | 8.35 | <0.05 | 0.2 | 93.9 | 2.3 | <2.1 | <0.05 | <0.05 | 90.5 |
| | Kiosk | <0.8 | <2.1 | <0.05 | <0.05 | <7.98 | <0.8 | <2.1 | <0.05 | <0.05 | <7.98 |
| Neosho, MO | Tap | - | - | - | - | - | - | - | - | - | - |
| | Kiosk | 5.35 | <2.1 | <0.05 | <0.05 | 56.8 | 3.9 | <2.1 | <0.05 | <0.05 | 53.5 |

**Table S11.** Plumbing-based metals and total phosphorus determined in kiosk samples during the Fall 2024 sampling campaign (unless otherwise noted). Numbers with a less than sign "<" denote samples below the method detection limit.

| Fall 2024 | | 1st Liter | | | | | 5th Liter | | | | |
|---|---|---|---|---|---|---|---|---|---|---|---|
| Location | Sample | Copper | Zinc | Tin | Lead | Total P | Copper | Zinc | Tin | Lead | Total P |
| | | µg/L | µg/L | µg/L | µg/L | µg/L | µg/L | µg/L | µg/L | µg/L | µg/L |
| Muscatine, IA | Kiosk – HP (11/2/24) | 142 | 170 | 0.7 | 1 | <7.98 | 72.8 | 83.1 | 0.08 | 0.39 | <7.98 |
| | Kiosk – HP (12/6/24) | 287 | 760 | 0.06 | 1.16 | <7.98 | 150 | 243 | <0.05 | 0.21 | <7.98 |
| | Kiosk – KI (11/2/24) | 158 | 144 | 0.14 | 1.1 | 315 | 239 | 13 | <0.05 | 0.15 | 305 |
| | Kiosk – KI (3/11/25) | 147 | 204 | <0.05 | 4.72 | <7.98 | 13.7 | <2.07 | <0.05 | 0.12 | <7.98 |
| Washington, IA | Kiosk | 3.61 | 141 | <0.05 | 0.25 | 318 | 1.34 | 69 | <0.05 | 0.19 | 360 |
| Baxter Springs, KS | Kiosk | 31.8 | 864 | 2.27 | 19.1 | <7.98 | 3.73 | 36 | <0.05 | 1.67 | <7.98 |
| Neosho, MO | Kiosk | 5.31 | <2.1 | <0.05 | <0.05 | 84.9 | 1.95 | <2.07 | <0.05 | <0.05 | 79.3 |
| Miami, OK | Kiosk | 2.56 | 6.01 | <0.05 | 0.08 | <7.98 | <0.8 | <2.07 | <0.05 | <0.05 | <7.98 |
| Rogers, AR | Kiosk | 46.88 | 184.81 | <0.05 | 0.30 | <7.98 | 24.42 | 45.48 | <0.05 | 0.19 | <7.98 |

**Table S12.** Summary of total samples and lead detections for each of the kiosks sampled across our study. Also provided are the highest lead concentration measured for each kiosk across all collected samples, and the amount of lead detected in ice dispensed from the kiosk.

| | | | No. of samples with lead greater than or equal to… | | | | | |
|---|---|---|---|---|---|---|---|---|
| **Kiosk** | **City, State** | **Total samples** | **Detection (>0.05 µg/L)** | **≥1 µg/L** | **≥5 µg/L** | **≥10 µg/L** | **Highest lead concentration (µg/L)** | **Lead in Ice** |
| Bettendorf (HP) | Bettendorf, IA | 3 | 3 | 1 | 0 | 0 | 1.07 | Not detected |
| Burlington (HP) | Burlington, IA | 2 | 0 | 0 | 0 | 0 | Not detected | Not detected |
| Clinton (HP) | Clinton, IA | 3 | 3 | 0 | 0 | 0 | 0.57 | Not detected |
| 53rd Street (HP) | Davenport, IA | 3 | 3 | 1 | 0 | 0 | 2.29 | 0.25 |
| Division St. (HP1) | Davenport, IA | 3 | 3 | 0 | 0 | 0 | 0.64 | Not detected |
| Des Moines (HP2) | Des Moines, IA | 3 | 1 | 0 | 0 | 0 | 0.07 | Not detected |
| Fairfield (HP) | Fairfield, IA | 3 | 0 | 0 | 0 | 0 | Not detected | Not detected |
| Fort Madison (HP) | Fort Madison, IA | 3 | 2 | 0 | 0 | 0 | 0.34 | Not detected |
| Moline (HP) | Moline, IL | 2 | 0 | 0 | 0 | 0 | Not detected | Not detected |
| Mount Pleasant (HP) | Mt. Pleasant, IA | 3 | 3 | 0 | 0 | 0 | 0.42 | Not detected |
| Muscatine (HP) | Muscatine, IA | 15 | 15 | 4 | 1 | 0 | **9.23** | Not detected |
| Muscatine (KI) | Muscatine, IA | 4 | 4 | 2 | 0 | 0 | 4.72 | 0.16 |
| Ottumwa (HP) | Ottumwa, IA | 3 | 1 | 0 | 0 | 0 | 0.06 | Not detected |
| St. Louis (HP) | St. Louis, MO | 2 | 0 | 0 | 0 | 0 | Not detected | 0.05 |
| Washington (HP) | Washington, IA | 4 | 2 | 0 | 0 | 0 | 0.25 | Not detected |
| Baxter Springs (KI) | Baxter Springs, KS | 4 | 4 | 4 | 1 | 1 | 19.1 | 0.08 |
| Joplin (TI) | Joplin, MO | 2 | 2 | 0 | 0 | 0 | 0.18 | 0.13 |
| Miami (TI) | Miami, OK | 2 | 1 | 0 | 0 | 0 | 0.08 | Not detected |
| Neosho (TI) | Neosho, MO | 4 | 0 | 0 | 0 | 0 | Not detected | 10.6 |
| Rogers (PS) | Rogers, AR | 2 | 2 | 0 | 0 | 0 | 0.30 | Not detected |
| | **Totals** | **52** | **45** | **10** | **2** | **1** | **19.1** | 10.6 |

**Table S13.** Paired total lead and dissolved lead (passing a 0.45 μm filter) on samples containing 1 μg/L or greater lead concentrations (excluding winter 2023 where a dissolved lead sample was not collected).

| Location | Date | Sample | Total Lead (μg/L) | Dissolved Lead (μg/L)[†] | Absolute Difference (μg/L)[a] |
|---|---|---|---|---|---|
| Muscatine, IA – HP | 09/18/2024 | 1St Draw | 1.10 | 0.98 | 0.12 |
| Baxter Springs, KS | 09/01/2024 | 1St Draw | 4.96 | 4.87 | 0.09 |
| Baxter Springs, KS | 09/01/2024 | 5th Liter | 1.54 | 1.56 | 0.02 |
| Muscatine, IA – HP | 11/02/2024 | 1St Draw | 1.00 | 0.68 | 0.32 |
| Muscatine, IA - KI | 11/02/2024 | 1St Draw | 1.10 | 0.63 | 0.47 |
| Baxter Springs, KS | 11/30/2024 | 1St Draw | 19.1 | 17.4 | 1.7 |
| Baxter Springs, KS | 11/30/2024 | 5th Liter | 1.67 | 1.67 | 0 |
| Muscatine – HP | 12/06/2024 | 1St Draw | 1.16 | 1.18 | 0.02 |
| Muscatine – KI | 03/11/2025 | 1st Draw | 4.72 | 5.06 | 0.34 |

[†]Dissolved lead analyzed after filtering past an 0.45 μm polypropylene syringe tip filter and acidifying to 2% nitric acid.
[a]Absolute difference = |total lead – dissolved lead|

**Table S14.** Spearman correlation summary for total metals (Cu, Zn and Pb) vs. water quality parameters. Water-quality parameters were measured at $5^{th}$-liter and applied to $1^{st}$ liter, $5^{th}$ liter, and pooled ($1^{st}$ + $5^{th}$) kiosk metals within site/campaign. Asterisks indicate $p$<0.05 (*), $p$<0.01 (**), and $p$<0.001 (***).

| Chemistry | Metals set | *n* | Spearman ρ | *p* value |
|---|---|---|---|---|
| Kiosk metals vs. kiosk pH | $1^{st}$ | 19 | -0.58** | $8.73 \times 10^{-3}$ |
| Kiosk metals vs. kiosk pH | $5^{th}$ | 29 | -0.67*** | $6.77 \times 10^{-5}$ |
| Kiosk metals vs. kiosk pH | All ($1^{st}$+$5^{th}$) | 48 | -0.63*** | $1.35 \times 10^{-6}$ |
| Kiosk metals vs. tap $H_2CO_3$* | $1^{st}$ | 15 | 0.77*** | $6.96 \times 10^{-4}$ |
| Kiosk metals vs. tap $H_2CO_3$* | $5^{th}$ | 15 | 0.84*** | $8.85 \times 10^{-5}$ |
| Kiosk metals vs. tap $H_2CO_3$* | All ($1^{st}$+$5^{th}$) | 30 | 0.80*** | $8.36 \times 10^{-8}$ |

**Table S15.** Correlation summary for lead only vs. water quality parameters. Water-quality parameters measured at 5$^{th}$-liter were applied to 1$^{st}$ liter, 5$^{th}$ liter, and pooled (1$^{st}$ + 5$^{th}$) kiosk lead within site/campaign. Asterisks indicate $p$<0.05 (*), $p<0.01$ (**), and $p<0.001$ (***).

| Chemistry | Metals set | *n* | Spearman ρ | *p* value |
|---|---|---|---|---|
| Kiosk Pb vs. kiosk pH | 1st | 19 | -0.64** | $2.95 \times 10^{-3}$ |
| Kiosk Pb vs. kiosk pH | 5th | 29 | -0.55** | $1.85 \times 10^{-3}$ |
| Kiosk Pb vs. kiosk pH | All (1$^{st}$+5$^{th}$) | 48 | -0.60*** | $6.92 \times 10^{-6}$ |
| Kiosk Pb vs. tap $H_2CO_3$* | 1st | 15 | 0.81*** | $2.26 \times 10^{-4}$ |
| Kiosk Pb vs. tap $H_2CO_3$* | 5th | 15 | 0.85*** | $5.85 \times 10^{-5}$ |
| Kiosk Pb vs. tap $H_2CO_3$* | All (1$^{st}$+5$^{th}$) | 30 | 0.81*** | $7.57 \times 10^{-8}$ |

**Table S16.** Plumbing-based metals and major cations determined in kiosk ice samples during the Summer 2024, Fall 2024, and Spring 2025 sampling. Numbers with a less than sign "<" denote samples below the method detection limit.

| Summer 2024 | | Ice Cations | | | Ice Metals | | | | |
|---|---|---|---|---|---|---|---|---|---|
| Location | Sample | $Na^+$ | $Mg^{2+}$ | $Ca^{2+}$ | Copper | Zinc | Tin | Lead | Total P |
| | | mg/L | mg/L | mg/L | µg/L | µg/L | µg/L | µg/L | µg/L |
| Clinton, IA | Kiosk Ice | 0.27 | 0.04 | 0.14 | 4.87 | <2.1 | <0.05 | <0.05 | <7.98 |
| Mt. Pleasant, IA | Kiosk Ice | 0.23 | <0.004 | <0.053 | 2.02 | <2.1 | <0.05 | <0.05 | <7.98 |
| Ft. Madison, IA | Kiosk Ice | <0.134 | <0.004 | <0.053 | 0.83 | <2.1 | <0.05 | <0.05 | <7.98 |
| Burlington, IA | Kiosk Ice | <0.134 | <0.004 | <0.053 | 1.2 | <2.1 | <0.05 | <0.05 | <7.98 |
| Muscatine, IA | Kiosk Ice - HP | 0.28 | 0.01 | 0.11 | 6.71 | <2.1 | <0.05 | <0.05 | <7.98 |
| Bettendorf, IA | Kiosk Ice | 4.42 | 2.02 | 3.27 | 17.6 | 7.67 | <0.05 | <0.05 | 57.38 |
| Davenport, IA - HP 1 | Kiosk Ice | 29.80 | 0.61 | 1.63 | 10.9 | <2.1 | <0.05 | <0.05 | 116.70 |
| Davenport, IA – HP2 | Kiosk Ice | 2.49 | 0.13 | 0.34 | 41.9 | 15 | <0.05 | 0.25 | <7.98 |
| Ottumwa, IA | Kiosk Ice | <0.134 | <0.004 | <0.053 | 1.09 | <2.1 | <0.05 | <0.05 | <7.98 |
| Moline, IL | Kiosk Ice | 11.26 | 1.06 | 6.77 | 18.1 | <2.1 | 0.12 | <0.05 | 13.75 |
| Fairfield, IA | Kiosk Ice | 0.39 | <0.004 | <0.053 | 0.87 | <2.1 | <0.05 | <0.05 | <7.98 |
| Washington, IA | Kiosk Ice | 22.71 | 0.31 | 0.64 | 4.34 | 8.63 | <0.05 | <0.05 | 67.55 |
| Des Moines, IA | Kiosk Ice | 1.07 | 0.04 | 0.12 | <0.8 | <2.07 | <0.05 | <0.05 | <7.98 |
| Baxter Springs, KS | Kiosk Ice | <0.134 | <0.004 | <0.053 | 1.34 | <2.1 | <0.05 | 0.08 | <7.98 |
| Joplin, MO | Kiosk Ice | 60.65 | <0.004 | <0.053 | 18.6 | <2.1 | <0.05 | 0.13 | 29.57 |
| St. Louis, MO | Kiosk Ice | 21.23 | 0.15 | 0.25 | 4.14 | <2.1 | <0.05 | 0.05 | 12.54 |
| Neosho, MO | Kiosk Ice | 33.34 | 0.00 | 0.06 | 161 | 121 | 0.71 | 10.6 | 21.31 |
| Fall 2024 | | | | | | | Ice Metals | | |
| Baxter Springs, KS | Kiosk Ice | 0.22 | 0.00 | 0.07 | <0.8 | <2.1 | 0.17 | <0.05 | 17.81 |
| Neosho, MO | Kiosk Ice | 16.19 | <0.004 | 0.13 | 8.89 | <2.1 | <0.05 | <0.05 | 12.25 |
| Rogers, AR | Kiosk Ice | <0.134 | <0.004 | 0.19 | <0.79 | <2.1 | 6.86 | <0.05 | 17.56 |
| Miami, OK | Kiosk Ice | 3.61 | 0.08 | 0.28 | 1.51 | <2.1 | <0.05 | <0.05 | <7.98 |
| Spring 2025 | | | | | | | Ice Metals | | |
| Muscatine, IA | Kiosk Ice – KI | 0.60 | 0.07 | 0.43 | 16.2 | 9.24 | 0.46 | 0.16 | 3.43 |

**Table S17.** Portable X-ray fluorescence results from Muscatine Kooler Ice and Highland Pure Water and Ice kiosks. Parentheses denote 2σ uncertainty of the measurement reported by the instrument.

| Sample | Location | % w/w Lead | % w/w Copper | % w/w Zinc | % w/w Tin | % w/w Nickel | XRF software alloy ID |
|---|---|---|---|---|---|---|---|
| Meter Nut | Muscatine - KI | 0.564 (0.02) | 65.5 (0.101) | 18.4 (0.076) | 0.642 (0.013) | 14.5 (0.06) | unknown |
| Meter Body | Muscatine – KI | 1.32 (0.026) | 63.3 (0.094) | 28.5 (0.073) | 0.736 (0.012) | 5.63 (0.033) | unknown |
| Coupling band | Muscatine - KI | 0.014 (0.005) | 0.319 (0.034) | 0.049 (0.011) | 0.024 (0.006) | 9.97 (0.117) | 316 Stainless Steel |
| | | | | | | | |
| PEX Tee (top rear) | Muscatine - HP | 0.172 (0.011) | 78.8 (0.087) | 20.9 (0.065) | 0.061 (0.006) | 0.017 (0.008) | C24000 Low Brass |
| PEX Elbow near pump | Muscatine - HP | 0.051 (0.007) | 76.3 (0.182) | 22.9 (0.088) | 0.034 (0.006) | < LOD | unknown |
| NPT Male fitting × PEX Compression fitting | Muscatine - HP | 0.025 (0.008) | 76.1 (0.126) | 21.4 (0.094) | < LOD | < LOD | C68700 Al Brass |
| Pressure reducing valve (water contact part) | Muscatine - HP | 0.137 (0.013) | 61.1 (0.13) | 36.3 (0.112) | 0.517 (0.017) | < LOD | C67500 Mn Bronze |
| PEX Tee (front left) | Muscatine – HP | 0.029 (0.007) | 74.2 (0.113) | 23.2 (0.083) | < LOD | < LOD | C68700 Al Brass |

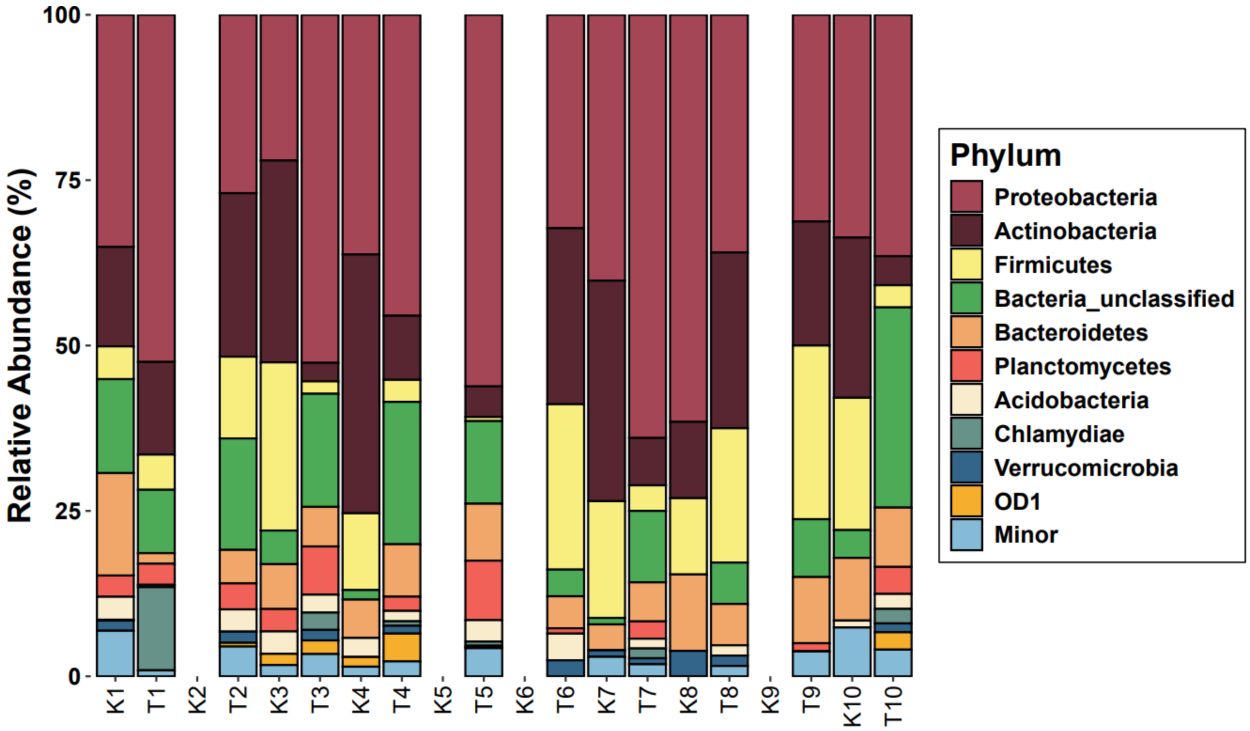


**Figure S1.** Relative abundance of bacterial phyla identified in kiosk and tap water samples from microbiome analysis. Microbiome analysis was not performed due to too low of a concentration of DNA from K2, K5, K6 and K9 samples. Locations: 1 – Des Moines; 2 – Clinton; 3 – Davenport HP1; 4 – Davenport 2HP; 5 – Bettendorf; 6 – Muscatine; 7 – Ottumwa; 8 – Mt. Pleasant; 9 – Fort Madison; 10 – Fairfield.

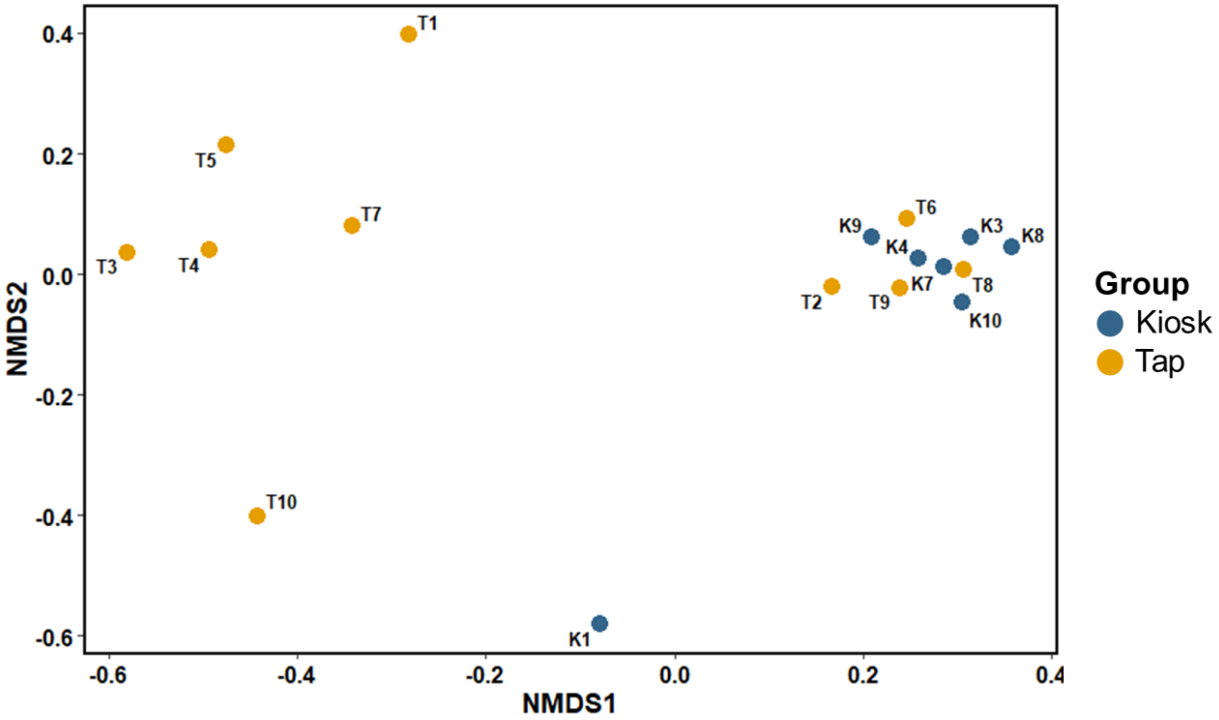


**Figure S2.** Bray-Curtis based NMDS plots showing difference between kiosk and tap water samples (Non-metric $R^2 = 0.996$, PERMANOVA test: p-value = 0.031 (*), Stress = 0.108141).

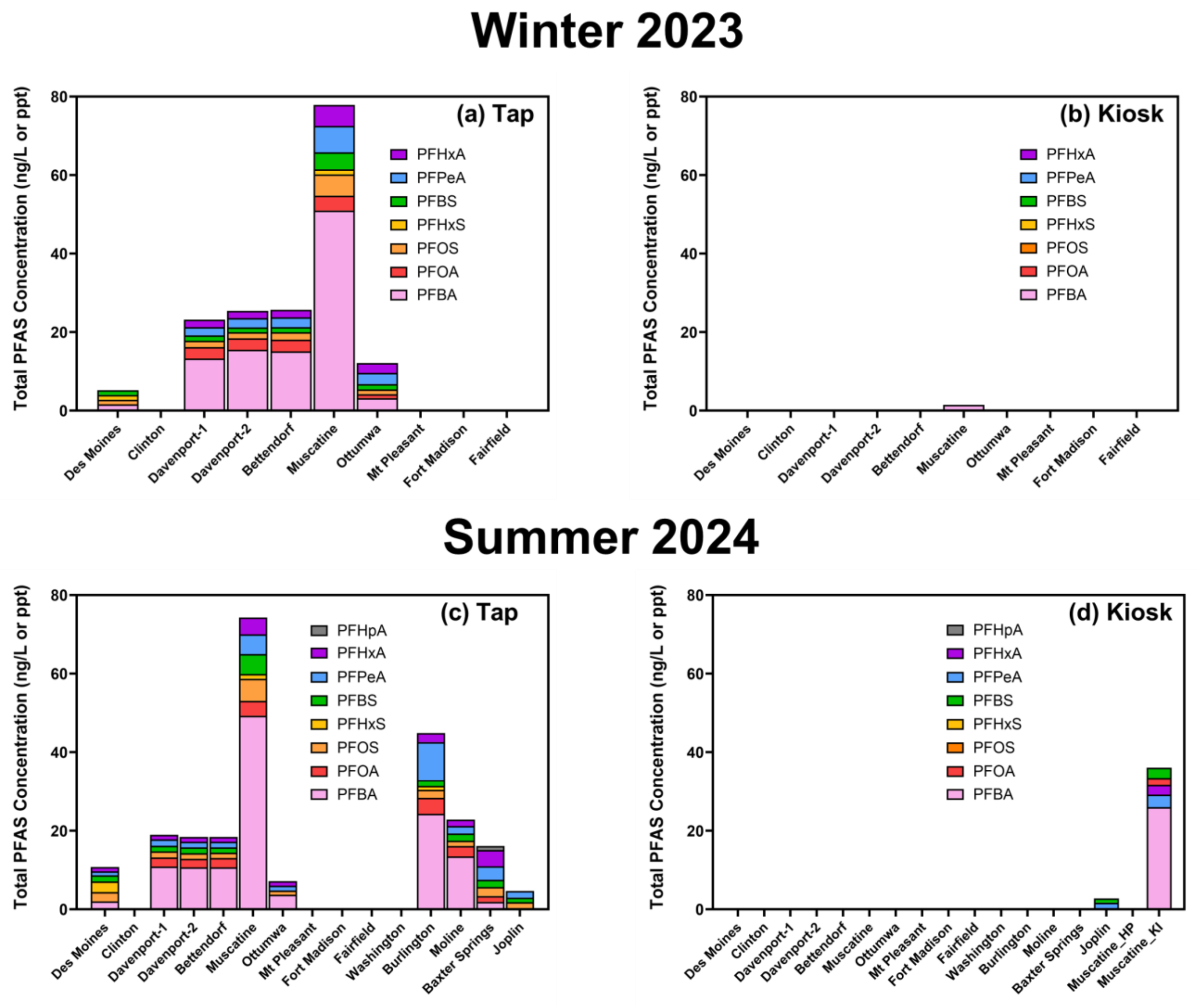


**Figure S3**. Total PFAS concentrations (in ng/L or parts per trillion; ppt) in paired (a) tap and (b) kiosk water samples collected in Winter 2023. Corresponding results for (c) tap and (d) kiosk water samples collected in Summer 2024 are also provided. The distribution of individual PFAS species detected in each sample is shown. If a location does not have any reported PFAS level, then the sample contained no PFAS species above our analytical method detection limit.

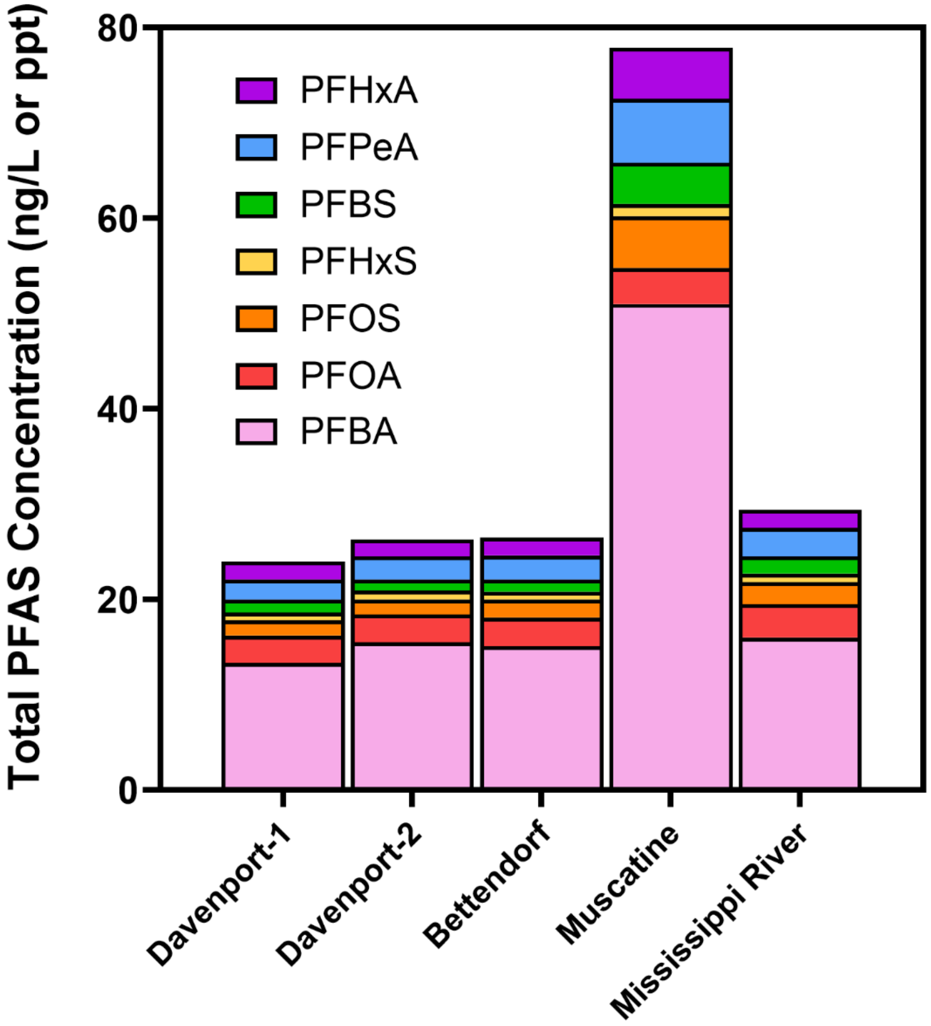


**Figure S4.** Total PFAS concentration, with species distribution, in tap water from Iowa cities relying on water either directly from (Davenport and Bettendorf) or impacted by (Muscatine) the Mississippi River. PFAS distribution in the Mississippi River, from a sample collected close to when paired tap and kiosk sampling was conducted in Summer 2024, is provided for comparison.

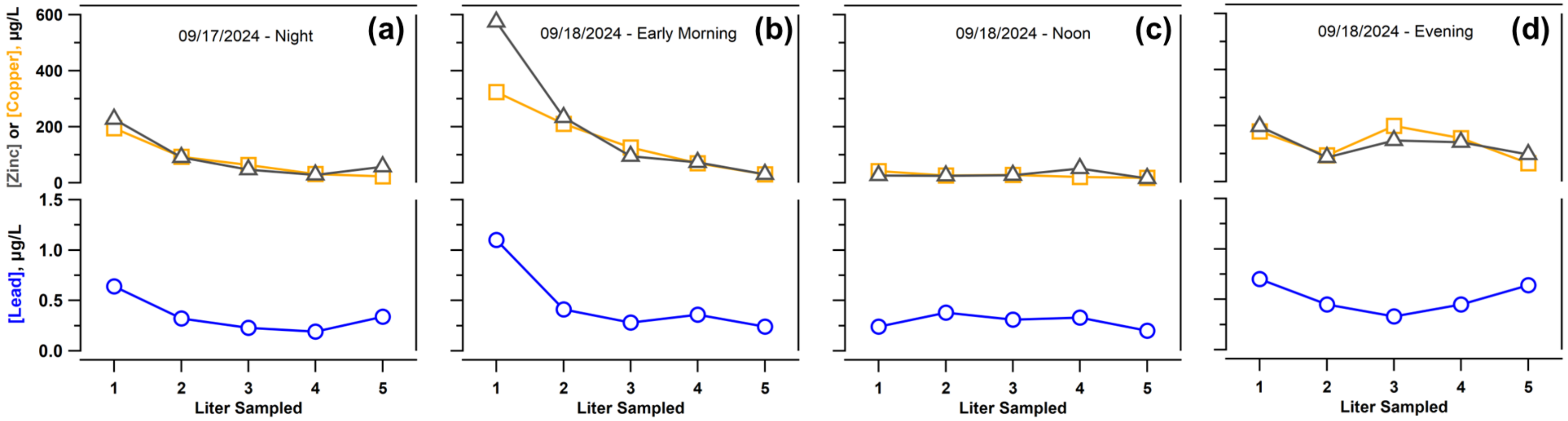


**Figure S5.** Temporal sampling of the HP kiosk in Muscatine, IA. Concentrations of lead, zinc and copper are shown for purchased kiosk water samples collected (a) at 10:30 PM the evening of September 17, 2024; (b) 5:30 AM the morning of September 18, 2024; (c) 12:00 PM on September 18, 2024; and (d) 6:00 PM the evening of September 18, 2024. Samples were collected and analyzed for each liter of kiosk water dispensed between the more conventional 1st liter and 5th liter sample.

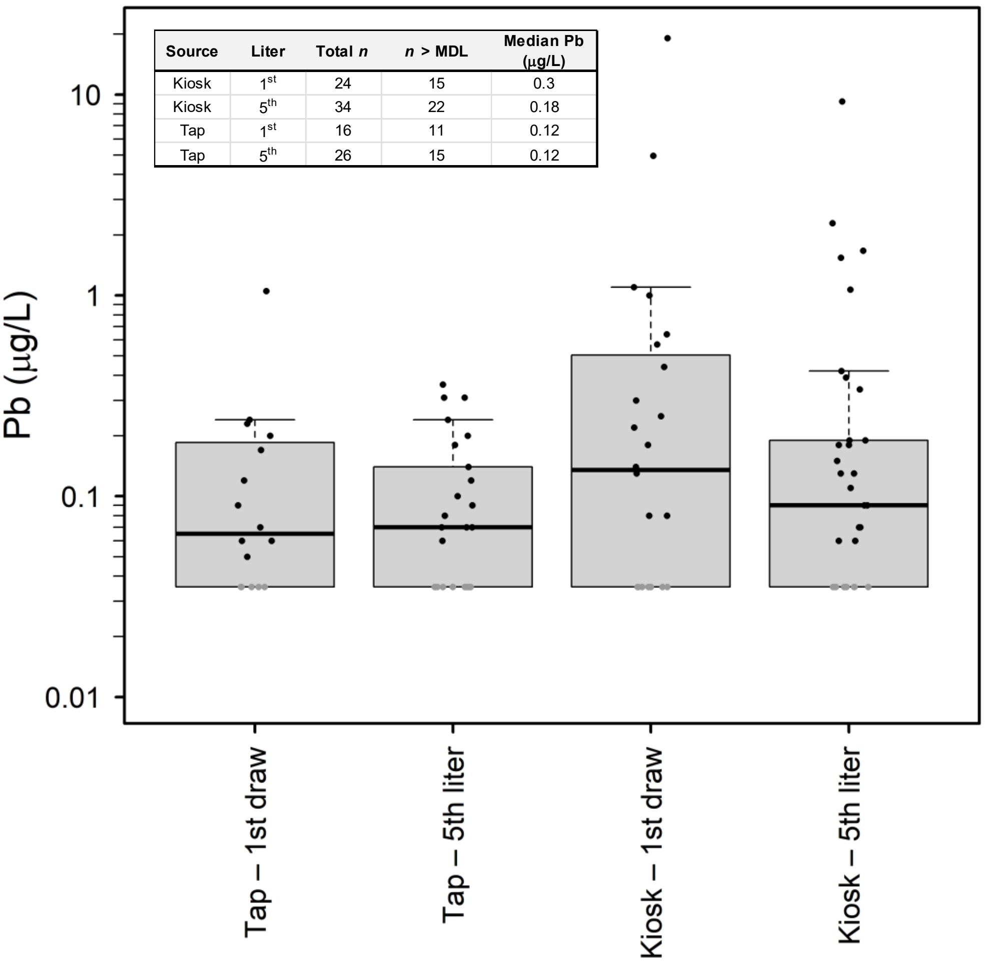


**Figure S6.** Box and whisker plot of tap and kiosk 1st draw and 5th liter lead concentrations (µg/L). Boxes show the interquartile range (IQR) and the whiskers show 1.5 × IQR. Lead concentrations below the MDL were set to MDL/√2 and are show as grey dots. A summary table with relevant data descriptors is included in the inset.

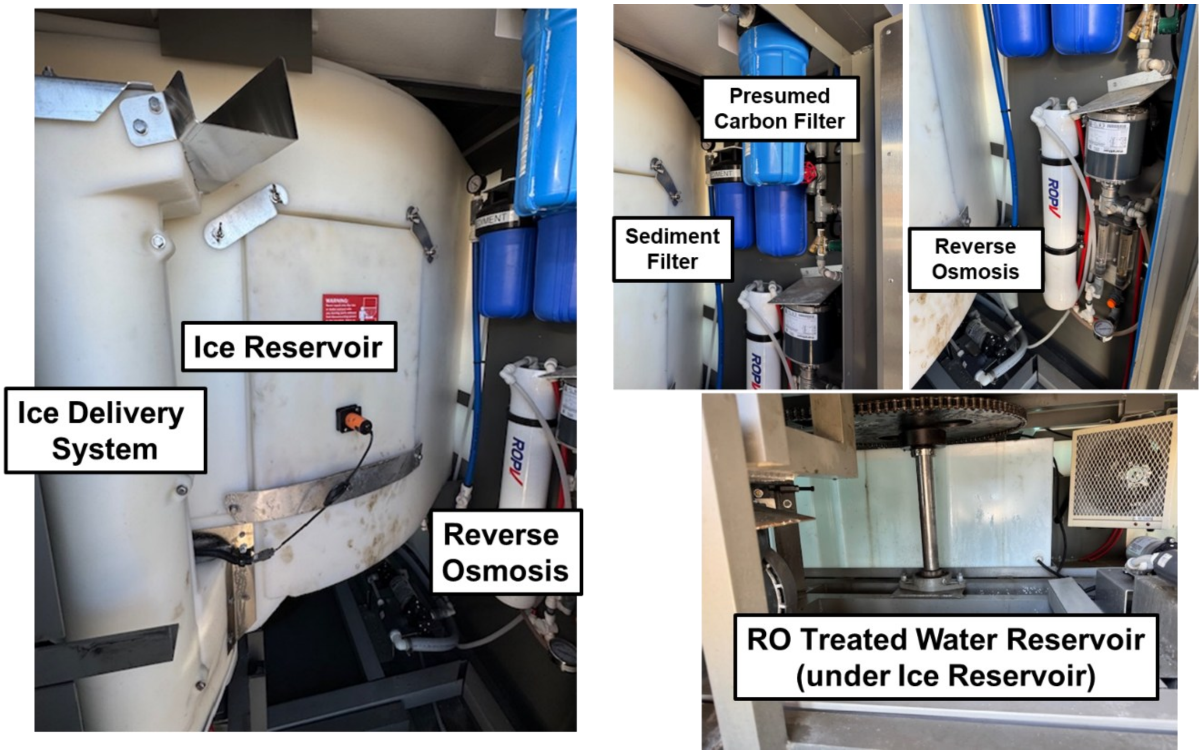


**Figure S7.** Pictures of the inside of a Kooler Ice kiosk. Key parts are identified.

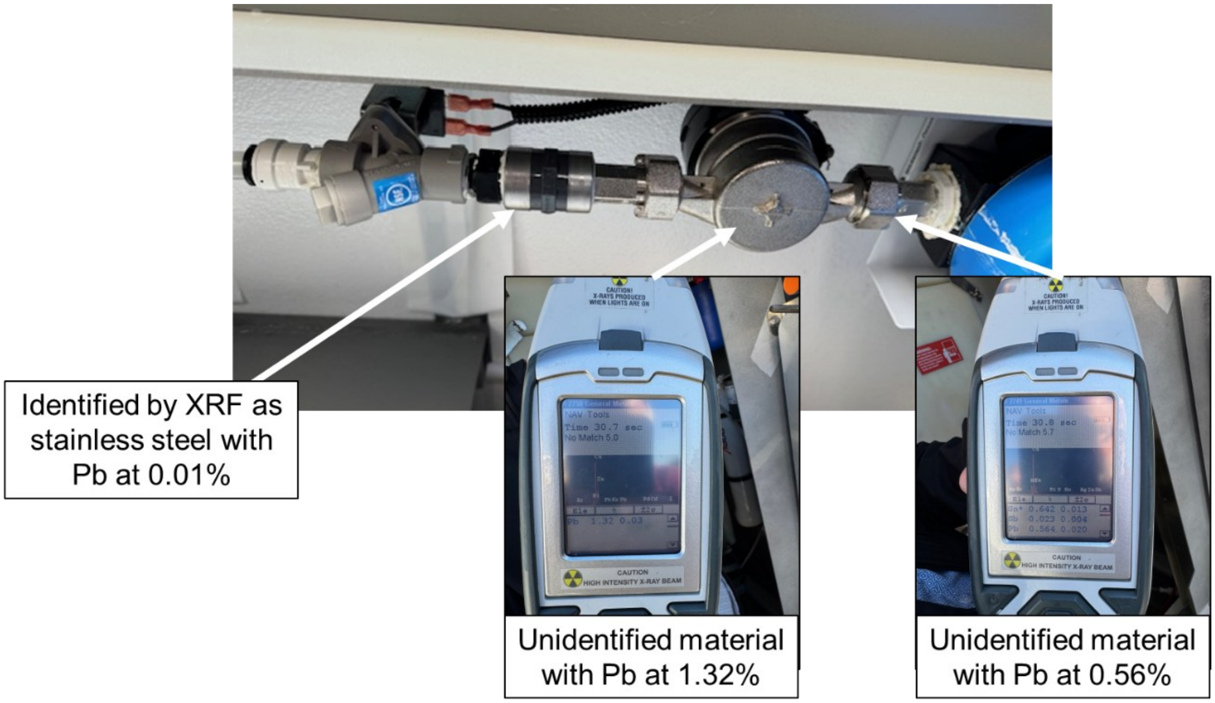


**Figure S8.** Metal water meter found in the KI kiosk, along with portal XRF results for lead.

## Supplemental References


1. L. Rosenblum, S. C. Wendelken, "Method 533: Determination of Per- and Polyfluoroalkyl Substances in Drinking Water by Isotope Dilution Anion Exchange Solid Phase Extraction and Liquid Chromatography/Tandem Mass Spectrometry" (EPA Document 815-B-19–020, United States Environmental Protection Agency, 2019); https://www.epa.gov/sites/default/files/2019-12/documents/method-533-815b19020.pdf.

2. Hach Company/Hach Lange GmbH., "Chlorine, Free - Method 8021" (USEPA DPD Method DOC316.53.01023, 2014); https://www.hach.com/asset-get.download-en.jsa?id=7639983694&srsltid=AfmBOooOPPkcJS9i_o1WKkURR5yQXjmsgrjDK3qqX_a4QqZcFQZ0Gi-e.

3. Hach Company/Hach Lange GmbH., "Chlorine, Total - Method 8167" (USEPA DPD Method DOC316.53.01027, 2022); https://www.hach.com/asset-get.download-en.jsa?id=7639983698&srsltid=AfmBOoopB546-uaavXNJEg_N_3qnct-jnI3RZnKoU9KgHirysOijKSZe.

4. S. K. Shahi, K. Zarei, N. V. Guseva, A. K. Mangalam, Microbiota Analysis Using Two-step PCR and Next-generation 16S rRNA Gene Sequencing. *J Vis Exp*, 10.3791/59980 (2019).

5. B. J. Callahan, P. J. McMurdie, M. J. Rosen, A. W. Han, A. J. A. Johnson, S. P. Holmes, DADA2: High-resolution sample inference from Illumina amplicon data. *Nat Methods* **13**, 581–583 (2016).

6. C. Quast, E. Pruesse, P. Yilmaz, J. Gerken, T. Schweer, P. Yarza, J. Peplies, F. O. Glöckner, The SILVA ribosomal RNA gene database project: improved data processing and web-based tools. *Nucleic Acids Research* **41**, D590–D596 (2013).

7. I. Vaz-Moreira, O. C. Nunes, C. M. Manaia, Ubiquitous and persistent *Proteobacteria* and other Gram-negative bacteria in drinking water. *Science of The Total Environment* **586**, 1141–1149 (2017).

8. P. Erdei-Tombor, G. Kiskó, A. Taczman-Brückner, Biofilm Formation in Water Distribution Systems. *Processes* **12**, 280 (2024).

9. K. To, R. Cao, A. Yegiazaryan, J. Owens, V. Venketaraman, General Overview of Nontuberculous Mycobacteria Opportunistic Pathogens: Mycobacterium avium and Mycobacterium abscessus. *Journal of Clinical Medicine* **9**, 2541 (2020).

10. V. Iliadi, J. Staykova, S. Iliadis, I. Konstantinidou, P. Sivykh, G. Romanidou, D. F. Vardikov, D. Cassimos, T. G. Konstantinidis, Legionella pneumophila: The Journey from the Environment to the Blood. *Journal of Clinical Medicine* **11**, 6126 (2022).

11. D. Wong, T. B. Nielsen, R. A. Bonomo, P. Pantapalangkoor, B. Luna, B. Spellberg, Clinical and Pathophysiological Overview of Acinetobacter Infections: a Century of Challenges. *Clinical Microbiology Reviews* **30**, 409–447 (2016).

12. D. Reynolds, M. Kollef, The Epidemiology and Pathogenesis and Treatment of Pseudomonas aeruginosa Infections: An Update. *Drugs* **81**, 2117–2131 (2021).

13. K. D. Brumfield, N. A. Hasan, M. B. Leddy, J. A. Cotruvo, S. M. Rashed, R. R. Colwell, A. Huq, A comparative analysis of drinking water employing metagenomics. *PLOS ONE* **15**, e0231210 (2020).

14. M. Park, S. Wu, I. J. Lopez, J. Y. Chang, T. Karanfil, S. A. Snyder, Adsorption of perfluoroalkyl substances (PFAS) in groundwater by granular activated carbons: Roles of hydrophobicity of PFAS and carbon characteristics. *Water Research* **170**, 115364 (2020).
